# Implementation of health and health-related sustainable development goals: progress, challenges and opportunities—a systematic literature review update

**DOI:** 10.1136/bmjgh-2025-021623

**Published:** 2026-02-02

**Authors:** Maya Kshatriya, Ruby Syal, Daina Als, Oviya Muralidharan, Busayo Akindole, Zahra A Padhani, Jai Das, Zulfiqar A Bhutta

**Affiliations:** 1 Centre for Global Child Health, The Hospital for Sick Children, Toronto, Ontario, Canada; 2 School of Public Health, The University of Adelaide, Adelaide, South Australia, Australia; 3 Robinson Research Institute, The University of Adelaide, Adelaide, South Australia, Australia; 4 Department of Paediatrics and Child Health, Aga Khan University, Karachi, Pakistan; 5 Institute for Global Health and Development, The Aga Khan University, Karachi, Pakistan

**Keywords:** Systematic review, Global Health

## Abstract

**Introduction:**

A prior systematic review assessed progress in health and health-related sustainable development goals (HHSDGs) from 2015 to 2019, identifying an important need for countries to strengthen implementation of multisectoral work, capacity building, financial stability and data availability. We undertook an updated systematic review to assess additional progress, challenges and opportunities for HHSDG implementation from 2019 to 2025, including the pandemic periods. This update aims to assess where countries are presently in HHSDG implementation and if further recommendations can be made in the final stretch to the 2030 targets.

**Methods:**

We followed a comparable comprehensive search strategy as the first review, focusing on implementation and acceleration strategies for HHSDGs. We undertook a qualitative synthesis from peer-reviewed and grey literature for specific databases, including studies and reports published from June 2019 to January 2025.

**Results:**

A total of 192 publications were included in the review of which 150 provided national-level information and 42 provided multicountry or regional information. Findings suggest a high level of political commitment in most countries and many HHSDG efforts being aligned with existing national development strategies. There was a noteworthy shift towards decentralised, subnational approaches to provide contextually relevant interventions. Multisectoral, multistakeholder, integrated approaches for implementation are increasing and proving to be effective. Diverse monitoring and evaluation strategies were employed, and (cross-country) knowledge sharing was instrumental to SDG policy and programme planning. Service disruptions incurred by the COVID-19 pandemic, lack of quality data and obtaining sustainable funding were frequently cited challenges to implementation.

**Conclusions:**

Ensuring continuous financial investments and strengthening data availability are essential to accelerate HHSDG implementation. Recommendations for progress include strengthening primary healthcare, fostering multisectoral collaboration and addressing deep-rooted societal perceptions around gender inequity. Future research should examine the interplay of multiple SDGs, and the impact of factors such as cost-effective cross-regional approaches for project implementation.

**WHAT IS ALREADY KNOWN ON THIS TOPIC**

- Health-related sustainable development goals (HHSDGs) are increasingly being aligned and implemented through multisectoral and multistakeholder initiatives.
- Ongoing challenges to implementation include limited financial resources, high donor dependency, inadequate mainstreaming of SDGs in subnational planning and budgeting and lack of disaggregated and reliable data.

**WHAT THIS STUDY ADDS**

- Notable emphasis on the use of cross-country knowledge sharing by means of turning to previous successful exemplars/strategies for informing HHSDG-focused policy and programmes.
- Greater increase in sharing experiences and models of implementation across countries and regions, allowing nations to apply potentially useful implementation approaches in their context.
- Increasing adoption of innovative approaches, such as the Baby-Friendly Hospital Initiative (SDG 1, 2, 3, 4, 5, 8 and 10) and the Global Action Plan for Healthy Living and Well-being for All implemented in the Kyrgyz Republic.

**HOW THIS STUDY MIGHT AFFECT RESEARCH, PRACTICE OR POLICY**

- Cross-country and cross-regional knowledge and resource sharing, spanning policy and programme levels, have been instrumental in advancing HHSDG programmes, with nations adopting and adapting successful strategies from other countries. This form of collaboration highlights the critical role of global partnership in achieving SDGs across low and middle-income countries (SDG 17).
- Future research could focus on examining the complexity and interplay of multiple SDGs, the impact of factors beyond the SDGs, as well as efficient and cost-effective cross-regional approaches for project implementation and monitoring, as well as linking information and datasets as a way of resource sharing.
- The impacts of poly-crises (climate change, conflict, political violence, epidemics/pandemics, etc) and the ability of states to reach the SDGs is another area that can be further examined.

## Introduction

 Five years remain in the ambitious plan for the sustainable development goals (SDGs). While the goals are categorised into 17 distinct thematic areas, the SDGs are closely interconnected. Good health is one clearly mentioned goal, while the other 16 goals address a range of determinants, which directly and indirectly impact health and related outcomes. Given the interconnectedness of the SDGs, ensuring progress requires an element of integrated implementation to achieve results across all goals. To address health-related sustainable development goals (HHSDGs), focusing on key social, economic and environmental dimensions of health is essential.

The previous 2020 review indicated a high level of political commitment in most countries.^1^ A multisectoral integrated approach was also adopted in institutional setups, but evidence of the effectiveness of these approaches was limited. Funding constraints remained a major challenge for many countries, with HHSDGs being financed within existing funded plans or through SDG-specific budgeting and tracking. Additional funding was mobilised by increasing domestic taxation and subsidisation, and by collaborating with development partners and the private sector. Evidence on equity promotion, capacity building initiatives and implementation approaches at subnational level was also limited, including lack of coordination among various levels of government.

This systematic review update assessed current evidence around whether: (1) countries are making commitments to national-level HHSDG implementation; (2) strategies for integrated implementation are being adopted, what those strategies are, and what early lessons are emerging at national and subnational levels and (3) gaps and challenges are evident in HHSDG implementation.

## Methods

### Study design

A systematic review methodology comparable to the previous review was applied to address our research question.^1^ This included reviewing all papers that examined the implementation of HHSDG initiatives at the national level and using select key sources for non-peer-reviewed literature. Studies from June 2019 to January 2025 were included.

### Search strategy and information sources

Medline, Embase, CAB Abstracts, CINAHL, Cochrane (CENTRAL Register of Controlled Trials and Database of Systematic Reviews), 3ie Databases of Impact Evaluations and WHO regional databases (WHOLIS) were searched for peer-reviewed literature (online supplemental table 1a). A comprehensive list of grey literature sources was also searched (online supplemental table 1b). The same Population, Intervention, Comparison and Outcomes elements for search terms were used as in the previous review (online supplemental table 2). Only English language publications were retrieved.

### Study framework of analysis

**Figure 1:**
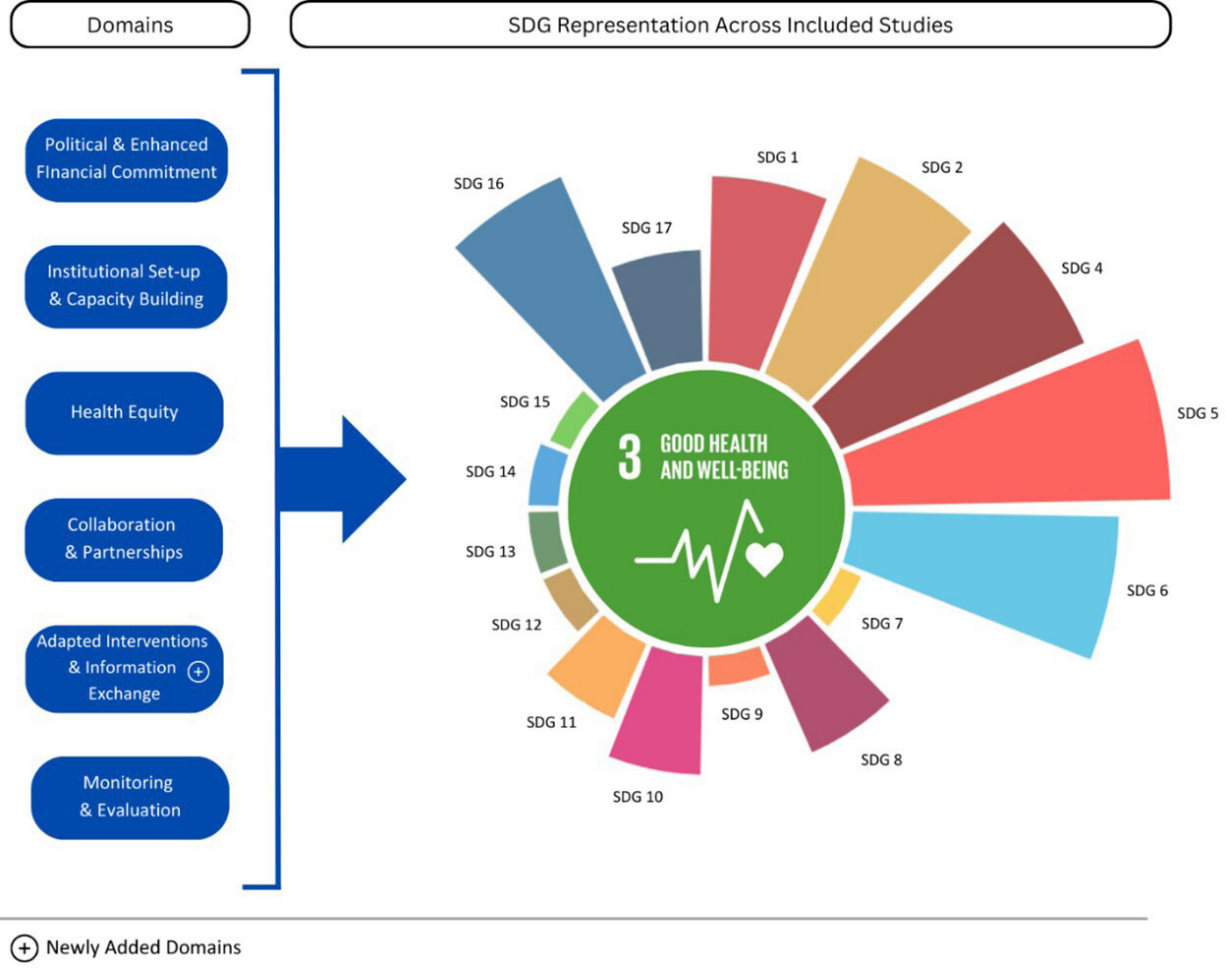
Revised analytical framework for the review. SDG, sustainable development goal.

A modified analysis framework was used, based on the previous review framework (figure 1). The same guide for screening, analysis and synthesis of literature was followed.

The previous framework incorporated various stages of policy implementation, ranging from generating political commitment to monitoring impact, building on existing Health-in-All-Policies frameworks. The nine domains identified in the initial review were regarded as representing essential processes for planning and implementing HHSDGs at the national level. These nine domains remained relevant in this review and have been integrated into a revised framework. Some domains were combined due to overlapping content and to enhance analytical clarity. The subdomain—evidence-sharing and adapted interventions—was added to capture the critical role of exchanging expertise and innovations across countries, particularly between LMICs, to strengthen SDG implementation. Collectively, the domains in this revised framework represent the political, technical and institutional determinants that influence progress towards HHSDG targets and indicators. The HHSDGs include SDG 3 (Good Health and Well-being) as well as select targets and indicators from the remaining 16 SDGs.

The framework outlines that high-level political commitment must be translated into impact through suitable institutional arrangements, adequately resourced programmes, meaningful stakeholder engagement and effective cross-sector collaboration to drive health and related outcomes. Continuous process monitoring is essential for evaluating effectiveness and impact. Furthermore, the framework acknowledges foundational values guiding public health policy, particularly the principle of equity.

### Eligibility criteria

The same inclusion criteria were followed from the previous review.^1^ Eligible publications discussed the implementation of HHSDGs at national and/or subnational levels. No restriction was applied to study design.

### Exclusion criteria

Publications that provided information at multicounty or regional levels without any national and subnational information, and for which disaggregated information was not available, were excluded. Publications only discussing normative guidance or possible approaches for HHSDG implementation were also excluded.

### Screening and data extraction

Four experienced reviewers independently screened title and abstract records for peer-reviewed articles or title and snippet for grey literature. Screening was done in duplicate, with discrepancies resolved through consensus. Full-text screening and data extraction for peer-reviewed articles were also done in duplicate. Given the volume of grey literature, initial title and abstract screening were done in singlet while extraction was done in duplicate. The same methodology for data extraction was used from the previous review, following a predesigned, tested form based on the initial analytical framework. A risk of bias assessment was not applicable as the studies included were not hypothesis-testing.

### Data synthesis and analysis

The Preferred Reporting Items for Systematic Reviews and Meta-analyses checklist was used as a reporting tool to be comprehensive and transparent (online supplemental table 3).^2 3^

Each publication was extracted and analysed using a prespecified analytical framework, along with key terms associated with each domain. Domains were evaluated independently and synthesised into relevant categories and themes. The categorisation of information and methods of analysis varied for each domain. For instance, the analysis of information exchange was stratified into distinct tiers, encompassing policy-level, programme-level and overall-level sharing (online supplemental table 4).

As a post-hoc analysis, actions were systematically extracted from included reports, and two independent reviewers grouped these studies to identify common areas of focus for country-level implementation of SDGs. The final categorisation was confirmed through consensus by the review team.

Challenges to HHSDG implementation across all studies were also thematically analysed and subsequently categorised in accordance with the domains of the prespecified analytical framework and in relation to the COVID-19 pandemic, across prepandemic, during and postpandemic periods (online supplemental table 5). A similar analysis was done for implementation strategies of HHSDGs across all studies (online supplemental table 6). For the prepandemic period, we assessed studies published since the previous review between July 2019 and February 2020. The pandemic was defined as March 2020 to May 2023, and the postpandemic period as June 2023 onwards in accordance with the WHO declaration that COVID-19 is no longer an international public health emergency.^4^,^6^

## Results

**Figure 2:**
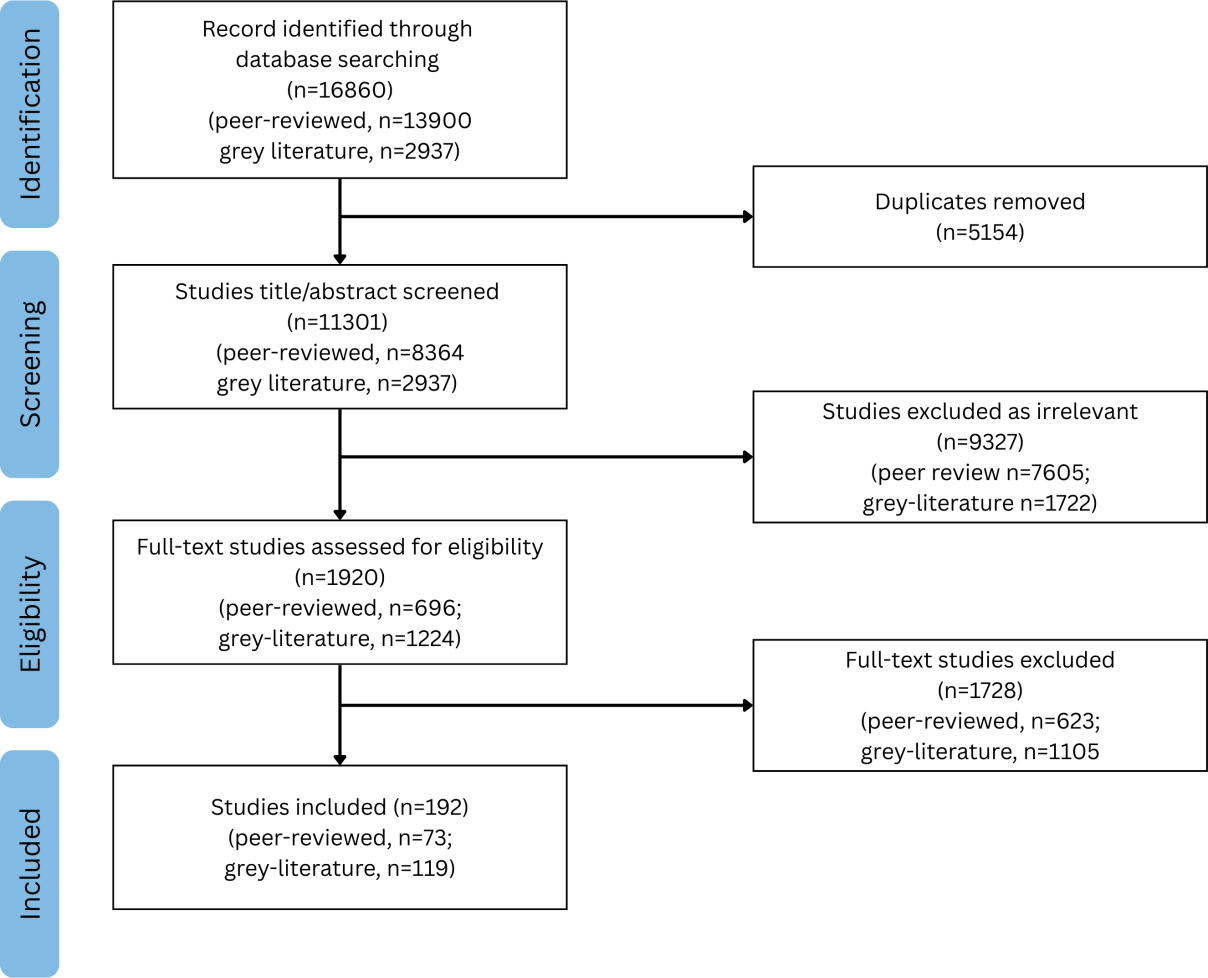
Flow diagram.

Of the 16 860 publications identified, 11 301 titles and abstracts were screened after deduplication. A total of 9327 publications were excluded by two independent reviewers based on the eligibility criteria. Of the 1920 full-text publications assessed, 192 were included in the final analysis, 73 peer-reviewed and 119 grey literature sources (figure 2).

### Characteristics of included publications

**Figure 3:**
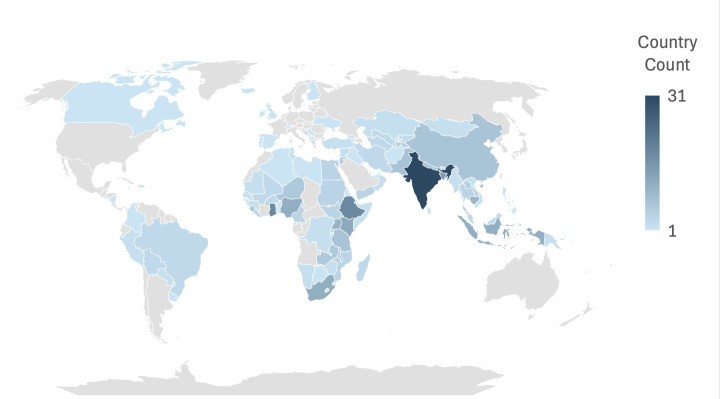
Geographical distribution of included studies.

Of the studies included, 150 had findings from individual countries and 42 provided multicountry information. Across all studies, Sub-Saharan Africa (SSA) was referenced 161 times, South Asia (SA) 61 times, East Asia and Pacific (EAP) 56 times, Europe and Central Asia (ECA) 39 times, Middle East and North Africa (MENA) 32 times, Latin America and the Caribbean (LAC) 17 times and the Americas 1 time (figure 3). Detailed characteristics and key findings of each publication are presented in online supplemental table 4.

The rising number of peer-reviewed publications on the SDGs showcases increased academic and global engagement with these goals. Peer-reviewed publications steadily increased from 2015 to 2024, with a notable rise in implementation studies at the onset of the pandemic. Additional information and figure are provided in online supplemental figure 1.

The top three most frequently cited SDGs across studies were SDG 3: Good Health and Well-being (n=109), SDG 5: Gender Equality (n=43) and SDG 6: Clean Water and Sanitation (n=36). Eighty-four studies discussed two or more SDGs, highlighting the interconnectedness of these goals and the importance of integrated approaches in addressing health challenges. Notably, SDG 13: Climate Action was cited four times, emphasising the need for increased attention to climate-related issues and their broader impact on health and sustainability. A table reporting the prevalence of each SDG across included studies is available in online supplemental table 7.

The included studies reported a wide array of strategies for addressing HHSDGs (table 1, online supplemental figure 2). Capacity-building initiatives were identified as the most extensively studied strategy, which included training health workers and establishing partnerships. These findings underscore the breadth of strategies reviewed, highlighting efforts across additional diverse sectors such as infectious disease management, urban planning and digital health initiatives to advance the SDGs.

**Table 1:** Number of studies grouped by intervention category

Category	Number of studies	Description of interventions included	SDGs																
Capacity building initiatives	36	Training of health workers (midwives, radiologists, nurses, speech-language pathologists), medical supply chain, health-system strengthening, partnerships, SDG coordination centres	1	2	3	4	5	6		8	9	10						16	17
Health equity programmes	27	Programmes targeting historically excluded groups, digital and financial inclusion	1	2	3	4	5	6		8		10						16	
Sexual and reproductive health programmes	22	Interventions addressing gender-based and intimate-partner violence, female genital mutilation, child marriage, HIV transmission and care, use of contraceptives	1		3	4	5			8		10	11		13			16	
WASH initiatives	15	Waste management, water services in rural areas,	1	2	3	4	5	6					11	12		14		16	
Community-based health programmes	15	Community-health worker programmes, community engagement and ownership and community-based programmes		2	3		5		7	8									17
Health insurance schemes	10	Free healthcare, minimum health packages			3		5												
Emergency resilience building	10	Pandemic preparedness, conflict, climatic shocks	1	2	3	4	5	6	7	8	9	10	11	12	13	14	15	16	17
Cash transfer programmes	10	Conditional and unconditional cash transfers, livelihood support, social protection programmes	1	2	3	4	5	6		8	9	10			13	14	15	16	
Early childhood development	9	Early childhood education centres and training parents			3	4	5			8		10							
Nutrition and food security	9	Policy development, public-private partnerships, efforts to support IYCF	1	2	3	4	5	6				10						16	17
NCD promotion	7	Programmes to promote physical activity		2	3														17
Digital health initiatives	6	Mobile and digital health services, e-health tools and systems for information management, ICT-enabled community health workers	1	2	3	4	5		7	8	9	10	11						17
Product regulations	6	Tobacco, salt and fat content, pesticides	1		3					8		10						16	
Urban planning	4	Sustainability considerations in urban planning		2	3			6				10	11						
Road safety measures	4	Regulations, information management systems and sensitisation programming			3								11						
Infectious disease management	3	AMR strategies and management of drug-resistance especially in the context of TB		2	3	4		6				10				14		16	17

Colour shades represent the corresponding Sustainable Development Goals and are not intended to imply any statistical significance.

AMR, antimicrobial resistance; ICT, information and communications technology; IYCF, infant and young child feeding; NCD, non-communicable disease; SDG, sustainable development goal; TB, tuberculosis; WASH, water, sanitation and hygiene.

**Figure 4:**
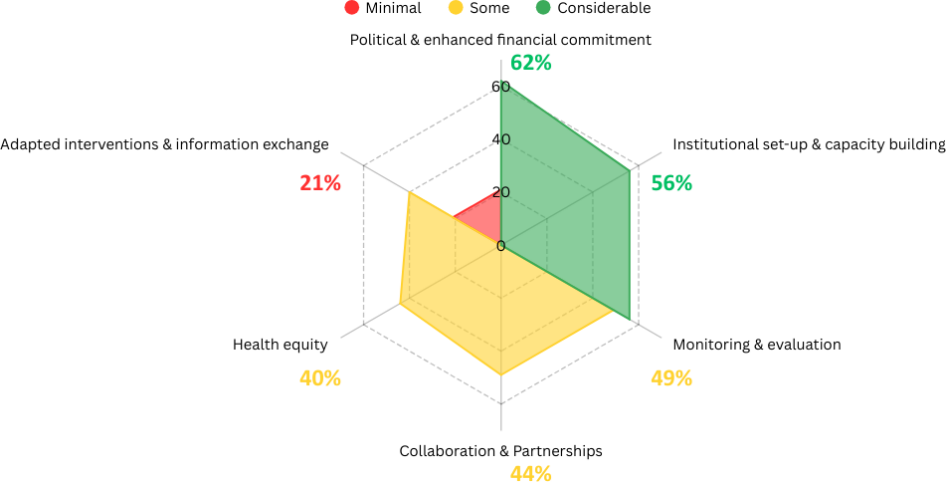
Availability of information about HHSDG implementation by subdomain for all included peer-reviewed studies. HHSDG, health and health-related sustainable development goal.

All findings were related and presented according to the six domains outlined in the revised framework. Figure 4 provides an overview of the available information across the six domains in all reports included. Further information on the process of generating this figure is available in online supplemental table 8. In addition to HHSDG-specific findings, evidence of implementation strategies that apply to SDGs generally, but are still pertinent to HHSDGs, was included. Regional and context-specific implementation strategies were also identified for specified HHSDGs, where possible.

### Political and enhanced financial commitment

Political commitment to the SDGs primarily presents as programming, policies, planning and strategies^7^,^9^ with greatest commitment at the national level, and some provincial, regional, local and international commitments.^10^,^12^ While the general population was more commonly addressed, women, children and refugees were noted as common subpopulations.

Policy implementation was found to require a multisectoral approach, with governments engaging NGOs, developmental partners, international entities and the private sector to achieve SDG targets.^13^ Legislative measures were also leveraged to provide a legal framework to support policy implementation and enforcement, addressing specific issues related to SDG 3 including child marriage, female genital mutilation (FGM) and access to health services, ensuring legal repercussions for non-compliance and that policies were effectively implemented.

Regional differences were noted, such as political commitment in EAP, MENA, SA and SSA focusing heavily on the health of women and children,^14^,^16^ and ECA focusing on achieving the SDGs and improving public healthcare.^7 17 18^

Financial commitment for SDG implementation came from various sources, including governmental bodies, international organisations (IOs) and private donors^919^,^27^ with main funders being national and IOs, such as UNICEF, WHO, World Bank and European Union, among others.^828^,^32^

The SDG Fund is one instance of funding allocation exclusive to the SDGs. This global fund was a collaborative initiative operating across joint programmes in 23 countries and engaging with UN agencies, governments, businesses and civil societies to advance the 2030 goals. The fund was utilised across several included studies, from investments to supporting household sanitation efforts in Ghana (SDG 6) to empowering vulnerable tea garden workers in Bangladesh (SDG 5).^1133^,^35^ Other instances of SDG-centred funding allocation were also reported. Cambodia’s progress towards meeting SDG 3 included the establishment of a Health Equity Fund to provide free healthcare access for poor populations.^36^ Ghana’s LEAP Program is another successful example of integrating programme implementation into the existing national health insurance scheme, promoting sustainability (box 1).^23^

Box 1 **Case study 1: Ghana LEAP Program**

Ghana’s advancements in achieving SDG 1, 2 and 3 include the Ghana Livelihood Empowerment Against Poverty (LEAP) Program, a flagship social protection initiative developed by the Ministry of Gender, Children and Social Protection (MoGCSP) to address extreme poverty. Launched in 2008, the programme provides bimonthly cash payments to extremely poor households, including those with orphans, vulnerable children, elderly without productive capacity, persons with severe disabilities and, since 2015, households with a pregnant woman or a child under 12 months. At the national level, the programme is managed by the Department of Social Welfare (DSW) under the MoGCSP. Ghana has 10 regions, each of which has its own LEAP directorate headed by the Regional Officer of the DSW. Furthermore, selected districts within the region have their own District Director to assist with implementation. Districts are selected by the District LEAP Implementation Committee. Finally, at the community level, beneficiary households are identified and selected by the Community LEAP Implementation Committee. The programme was also integrated into the existing National Health Insurance Scheme (NHIS), to ensure sustainability and broader reach.^23^

As of December 2017, the LEAP Program reached over 213 000 extremely poor families across Ghana and was scaled up to enrol 350 000 households (1.5 million individuals) by the end of 2021. The programme has demonstrated success in increasing overall consumption, reducing poverty, improving food security, boosting NHIS enrolment, improving antenatal care, breastfeeding and preschool enrolment. The programme was also found to reduce intimate partner violence and increase support among women.^23^

According to a 7-year evaluation conducted by UNICEF, the Ghana LEAP Program contributed to notable improvements in the health-related sustainable development goals (HHSDGs) and their targets. By 2022, the programme led to a 3.1 percentage point reduction in poverty and a 5.7 percentage point decrease in extreme poverty, directly supporting SDG 1.2 (reducing poverty in all its forms). Additionally, LEAP 1000 significantly increased the probability that children ever attended school and improved literacy, with statistically significant gains observed among older children (12–17 years old) and girls, aligning with SDG 4.1 (universal primary and secondary education) and SDG 4.6 (youth literacy and numeracy). These outcomes reflect the LEAP programme’s impact on improving social determinants of health and advancing progress towards key HHSDG targets.^144^

Challenges still exist around SDG funding such as insufficient and inconsistent funding,^22 37^ inadequate planning and cost estimations, differing levels of local government capacities, lack of resources in remote and rural settings^36 38^ and political downturn, resulting in anticipated funding not being received.^39^ Other challenges include over-reliance on government budgets, external funds and/or out-of-pocket expenditures for programmes to continue operating.^22 40 41^

Recommendations to reach the ambitious targets of the SDGs included expanding existing national financing schemes,^42^ encouraging governments to provide subsidies^43^ and increasing financing for health from government and donors.^27^

### Institutional setup for multisectoral programmes and capacity building

The implementation of SDG programmes and policies was largely achieved through context-specific strategies and establishing key stakeholder support. Ministerial bodies, including MoH, Education and Development, frequently lead national SDG-related programmes or policies.^44 45^ Humanitarian organisations and their subsidiaries, such as UNICEF country and regional offices, played a pivotal role in guiding and driving national-level SDG initiatives, especially in low-resource settings characterised by political instability and a heavy reliance on aid organisations for essential services. As noted in the original review, several countries established new structures or frameworks to address SDG-related objectives;^39 46^ however, many studies in this updated review leveraged existing structures, institutions and systems.^47^,^49^ The Tajikistan 2021–2030 National Programme aims to build on the existing health financing system to improve the efficiency of healthcare service provision, while addressing added concerns related to equity, accessibility and affordability of primary healthcare (PHC) and other essential health and social services.^49^

While primarily implemented at the national level, subnational programmes and policies were also identified, offering a decentralised approach where interventions were contextually relevant.^25 50^ To address the challenge of limited knowledge and understanding of water, sanitation and hygiene (WASH) issues and management in certain regions of Cambodia, a subnational strategy was developed, including a WASH governance guide.^50^ This subnational guide was derived from the national one, undergoing revisions based on feedback from local teams and multi-stakeholder consultations.^50^ In this case, the decentralised reform shifted the decision-making power to the people who best understood the issues facing their regions.

There was an emphasis on strengthening PHC services. As the first point of contact for many individuals within health systems, integrating services within PHC supports universal health coverage (UHC) (SDG 3.8). A study by Hanafiah *et al*^51^ examined Malaysia’s Enhanced Primary Health Care intervention as part of the country’s commitment to SDG 3.8. The intervention aimed to provide comprehensive, patient-centred care and systematically address non-communicable disease (NCD) management at the primary care level via prevention, early detection and treatment.

Capacity development activities prioritised knowledge and skill building, resilient infrastructure development, technology integration and policy and strategy alignment. With a focus on SDG 3 and 4, training programmes and workshops aimed to enhance knowledge, competencies and specialised skills of health professionals and community members,^52^,^55^ with several of these programmes undergoing an evaluation to assess impact. As part of a PHC initiative in rural Ethiopia, midwives underwent obstetric ultrasound training and hands-on learning, leading to possible prevention of 1970 maternal morbidities and mortalities per 100 000 live births.^52^

Capacity-building programmes also placed emphasis on building resilient infrastructure, focusing on SDG targets 3.3 (end of epidemics and other communicable diseases) and 3.8 (achieving UHC). Interventions such as a multimodal biomedical waste management system in healthcare facilities across Bangladesh exemplified this effort.^20^ Public–private partnerships played a key role in delivering public health services as seen in Mozambique where UNICEF and UNFPA collaborated through a national partnership to end child marriage. This partnership built capacity by coordinating provincial level activities with local authorities and civil society organisations that supported adolescent girls’ rights (SDG 5.3: eliminate all harmful practices).^56^

Capacity development also played a pivotal role in shaping and implementing policy through developing comprehensive data systems,^57^ policy guidelines,^53^ feedback integration^51^ and identifying and addressing policy gaps.^58^ In Iran, the National Surveillance for NCD risk factors enabled policymakers to identify evidence-based, targeted interventions to address major health problems subnationally and allocate resources more effectively.^57^

The emphasis on knowledge building for parliamentarians and enhancing capacities of health sector workers in the previous review evolved to encompass broader sectors and more hands-on training initiatives for the health sector with a shift towards identifying and turning capacity gaps into action.

### Improving health equity

Countries are making specific efforts to identify and prioritise equity-deserving groups, such as women and girls (eg, pregnant women, mothers and adolescent girls), orphans, impoverished or low-income populations, people with disabilities, elderly and refugees and displaced populations.^16 24 29 56 59 60^ Common SDG targets tied to improving health equity among vulnerable populations include maternal mortality (3.1), neonatal mortality (3.2) and achieving UHC (3.8).^2833 61^,^63^ Many interventions focused on providing equitable and inclusive access to social protection, healthcare and educational programmes to improve literacy rates.^16 56 64^ Other interventions included promoting financial equity by creating employment opportunities or offering subsidies.^2933 56 65^,^69^ Some studies demonstrated success in improving equity using a community-based approach (box 2). In Bhutan, early childhood education centres have been effective for reaching marginalised communities in remote rural areas.^15^

Box 2 **Case study 2: a project to reduce maternal mortality in Sub-Saharan Africa through Information, Education and Communication (IEC) activities**

Senegal notably lagged behind other countries in reaching Millennium Development Goal (MDG) 5, which set a target of reducing maternal deaths to 170 per 100 000 live births. Launched in 2018 by Plan International Senegal and Plan International Korea, the Maternal Health Improvement Project aimed to decrease maternal and child mortality through improving health service quality, access and reproductive health awareness. This was a 3-year initiative that was developed in alignment with Senegal’s National Health Development Plan and was focused on reaching the remote districts of Sakal, Keur Momar Sarr and Coki through IEC strategies including home visits, village-based talks and radio programmes, towards achieving SDG 3 and reducing maternal mortality in sub-Saharan Africa.^60^ The project engaged a number of diverse actors, including Plan International, health facility staff, community-based organisations, local leaders and both male and female community members.

The programme was developed in alignment with national priorities and implemented by both local and international partners. Key achievements included the deployment of 1520 IEC campaigns, 10 016 home visits and nearly 9200 small-group village talks. The radio shows were conducted by midwives and had a focus on reproductive health. These activities saw a direct engagement with over 292 000 residents that were carried out by 37 community-based facilitators and 22 project assistants.

Looking specifically at the impact of these IEC activities on SDG outcomes (SDG 3.1, reducing maternal mortality), male awareness of ≥3 pregnancy danger signs improved significantly from 14.2% to 20.9% (p=0.029). Husbands who accompanied women to their antenatal care (ANC) visits increased from 14.7% to 22.3% (p=0.010). Measures of women’s empowerment also improved, with an observed increase in decision-making on one’s own health and participation in community decision-making groups. Additionally, male opposition to contraception declined from 34.4% to 23.7% (p=0.001), supporting progress towards SDG 5.6 (ensure universal access to sexual and reproductive health and reproductive rights).

Several challenges in the implementation of the programme were also noted. In Senegal, there is a notable emphasis on gender norms, which limited male participation and women’s autonomy. Among the individuals who participated in the project, there were high illiteracy rates (65.2% in 2018 and 53.3% in 2019), which could have affected health service uptake. Instead of appropriate facility deliveries, home deliveries were also reported due to a lack of means of transportation, remoteness of facilities and insufficient time to reach the health facility.

Overall, a short-term education campaign using IEC strategies was found to improve outcomes related to SDG target 3.1. There is a potential to implement these in other regions of Senegal and West Africa; however, special attention and adaptation would be required to implement them in East Africa where there are different cultural norms, societal expectations, religions and states of healthcare delivery systems that must be considered in order to tailor the intervention to those specific regions.

### Collaboration and partnerships

Multilateral collaboration was primarily reported among MoH, WHO, UNICEF and UNFPA.^17 30 70^ Common subthemes within the SDGs that involved these collaborations included children’s health, FGM, adolescent health and gender equity.^16 33 39 71^

Regional variations in priority-setting were noted for multisectoral collaboration(s).^1630 33 35 71^,^78^ In SSA, WHO, UNICEF, UNFPA and MoHs were active players and engaged in multilateral collaboration primarily focused on reducing gender-based violence (GBV) coupled with other health and health-related programmes.^16 30 33^

Multilateral collaborations mainly involved governments engaging with IOs for technical and financial support, or community and local governments to promote contextually relevant interventions. For example, the Turkish MoH partnered with the WHO to ensure Syrian refugees had access to quality health services addressing cultural and linguistic barriers and providing free, equal-standard care.^8 32 79^ Another study highlighted Tanzania’s National Sanitation Campaign, where the MoH and Social Welfare mobilised rural households to improve latrines and sanitation facilities, focusing on community-led total sanitation (CLTS).^21^

The pathways to meeting SDG targets require engaging and establishing partnerships at varying levels, both locally and internationally. Non-governmental organisations (61 studies) and UN agencies (62 studies) were the most active stakeholders. Among UN agencies, UNICEF (50 studies) was a primary stakeholder, engaging with local community partners, governments, public and private sector organisations to implement interventions or support existing structures.

Multisectoral government engagement was commonly reported, including different agencies within the government structure, with the MoH^1416 21 22 25 26 38 40 41 52 58 79^,^88^ and Ministry of Education^25 45 86 89 90^ being most reported.

Strategic engagement of specialised stakeholders was also observed. This included the involvement of individuals and groups such as religious leaders and community health workers to address targeted issues (eg, SDG 5.3, aiming to eliminate all harmful practices, such as child, early and forced marriage and FGMs) and promote societal change.^16 70^

Key development partners driving the SDG agenda include the UN (mainly UNICEF,^90^,^99^ UNFPA,^100^ UNDP^101 102^ and WHO), alongside governments and ministries.^7 44 73 87^ Primary roles included programme implementation, policy development, funding/budget allocation and capacity building.^7 62 103 104^ Regional differences included civil society organisations playing a greater role than NGOs in EAP,^55 87 104^ whereas in ECA, government actors were more involved.^7 11 62^

### Adapted interventions and information exchange

The exchange of knowledge, resource sharing and leveraging strategies from other countries has been crucial to the success of HHSDG programmes and policies.

At the policy level, expedited policy approval for tackling NCDs was achieved, as in the case of Iran, through high-level government involvement, coalition of research networks and leveraging experiences from other successful countries.^57^ Global policy dialogues like the Africa-China Poverty Reduction and Development Conference, led by UNICEF, advanced discussions on child poverty and supported African countries lagging in SDG progress.^55^

At the programme level, knowledge sharing was key for maternal, newborn and child health (MNCH) and WASH interventions. The success of Mexico’s conditional cash transfer (CCT) programme ‘Progresa/Oportunidades’, inspired a similar initiative in Odisha, India, offering financial incentives directly to women to promote behaviours that improve MNCH.^105^ Furthermore, Indonesia’s national sanitation programme, aiming for universal access to basic sanitation and safe water, mirrored the efforts of India’s Swachh Bharat programme, Bangladesh’s CLTS programme and Thailand’s safe sanitation initiative.^38^

Additional forms of knowledge and resource exchange included sharing technical expertise to build capacity. For example, UNICEF China established a training hub for MNCH, to share experiences with other developing countries.^55^ Financial support was secured through UNICEF China’s advocacy for vulnerable children in Malawi, Mozambique and Zimbabwe, indicating resource sharing at the policy level.^55^ Partnerships for SDGs have been strengthened, such as UNICEF’s collaboration with the Asian Infrastructure Investment Bank on WASH projects in Bangladesh, India and Pakistan.^55^ These findings highlight the significance of global collaboration and knowledge sharing in advancing HHSDG implementation across LMICs.^15^

### Monitoring and evaluation

Diverse strategies for monitoring SDG implementation and progress have been employed in programmes across LMICs. Common monitoring mechanisms include conducting home and field visits,^2223 26 27 36 38 43 48 53 105^,^111^ use of electronic applications (eg, mobile phones),^2223 38 48 53 59 60 65 105^,^107^ establishing local monitoring committees^51 60^ and tailoring monitoring tools (box 3).^24 112^

Box 3 **Case study 3: MomConnect—Pioneering Sustainable mHealth in South Africa**

Launched in 2014, MomConnect is South Africa’s pioneer national-scale digital health initiative that aims to improve maternal and child health outcomes by improving antenatal services on a national level. Through its comprehensive monitoring mechanisms, including user subscription, feedback via SMS and service ratings, MomConnect facilitates health communication and continuous improvement of healthcare services that are scalable and sustainable. The programme is currently ongoing and has three key features, including (1) registration of pregnant women into a national pregnancy registry, (2) weekly educational text messages and (3) an interactive help desk.^145^

After a decade of implementation and multiple evaluations, including randomised control trials and impact evaluations, MomConnect has demonstrated a positive impact on health behaviours and has demonstrated positive contributions to the health-related sustainable development goals (HHSDGs).^146^ Over the course of operations, the programme has reached over 5 million pregnant women and mothers, scaled to over 95% of public health facilities, all free of charge.^146^ Most notably, the programme has supported SDG Target 3.1 (reducing the maternal mortality ratio) by significantly increasing antenatal care (ANC) attendance, with 96% of mothers surveyed in 2024 attending at least four ANC visits, with an average of 7.3 visits. This was up from 76% in 2016.^147^

MomConnect has also demonstrated notable contributions to advancing SDG Target 3.b (essential medicines and vaccines), by supporting increases in early childhood immunisation rates. For instance, 89.9% of infants whose mothers used MomConnect received all six recommended vaccines at 6 weeks old.^147^

Additionally, a 2022 impact evaluation report found that women exposed to MomConnect messaging and were pregnant for the first time were more likely to breastfeed than women who were not exposed (p=0.013). Notably, a greater proportion of younger women (18–24 years old) who received the intervention (87.5%) breastfed at endline, compared with the control group (78.6%), who were not exposed to MomConnect. These improvements in breastfeeding practices and knowledge contribute to SDG Target 2.2 (ending all forms of malnutrition) and SDG Target 3.2 (ending preventable deaths of newborns and children under 5 years of age), as breastfeeding is a proven intervention for reducing child mortality and improving child health.^148^

The same 2022 impact evaluation found that in terms of reproductive health, family planning uptake increased to a greater extent between baseline and 8–10 weeks postpartum (72.7% to 89.5%, a 17% increase) in the group who received MomConnect compared with the control group (75.9% to 88.8%, a 10% increase).^148^ First-time and young mothers also reported significantly higher family planning self-efficacy scores postpartum.^148^ These results demonstrate notable contributions to SDG Target 3.7 (universal access to sexual and reproductive healthcare services, including for family planning, information and education).^148^

A stakeholder perspective analysis also identified a few challenges that were encountered in the implementation of the programme, including the need for expanded implementation and addressing challenges such as mismanagement, insufficient impact evaluations, data management, lack of leadership and support, unsuitable technology and over-dependency on external funders.^61^

Overall, MomConnect’s national mHealth approach has led to measurable improvements in ANC attendance, immunisation, breastfeeding and family planning. The programme’s impact is supported by robust evidence, demonstrating its value as a scalable, sustainable digital health intervention in South Africa that is contributing to advancements in the HHSDGs.

An additional strategy across all regions was the establishment of expert committees to oversee SDG implementation, with skilled committee members recruited from organisations including UN agencies, government entities, IOs and NGOs.^712 15 16 19 27 35 39 44 46 54 55 59 62 63 67 68 70^,^72 74^

There were notable differences in how monitoring activities were conducted across regions. In SSA, in-field data collection, conducted via home and field visits, was frequently utilised, along with monitoring committees that placed an emphasis on feedback sharing and discussed strategies for future improvement.^46^ Several countries also aimed to assess factors such as compliance.^16 65 125^

In LAC and ECA, public sources were often leveraged, consulting data from the Demographic and Health Surveys (DHS), Multiple Indicator Cluster Surveys (MICS) and national census data.^11 17 18 35 54 74 75 80 103 108 116 126^ In SA, tailored monitoring tools and systems were developed for programme and policy implementation.^70 77 86 105 119 122^ In India, a community-based monitoring mechanism was set up in 300 households to assess the impact of COVID-19 on vulnerable families. UNICEF India developed and established a result assessment module, known as RAM-India, for monitoring and reporting.^77^

Several countries noted challenges with conducting monitoring initiatives, such as the COVID-19 pandemic, which prevented field visits as originally planned.^119^ Among vulnerable populations, there were also difficulties ensuring the availability of high-quality, timely health information, and clarity on indicators for effective monitoring.^14 108 127^

Similar to the original review, evaluation activities have been underway in most countries, mainly to assess SDG progress. Common data collection tools included questionnaires, surveys, interviews, facility data, logbooks and field observations.^60 85 105^ National surveys such as the DHS and MICS were frequently used to assess SDG indicators over time;^16 37 46 56 83^ however, lack of adequate funding for the DHS in regions like Nigeria hindered policy implementation.^37^ In some instances, relevant stakeholders that were part of policy and programme implementation were often actively engaged in evaluation activities (eg, in-depth interviews) to better understand programme effectiveness.^128^

Evaluation strategies across most studies aimed to assess how health and related programmes and policy implementation affected key populations, including women and children, with less consideration given to individuals with disabilities.^1435 70^,^72 82 114^

Regions like SSA and SA have a strong emphasis on community involvement in evaluation, highlighting the role of grassroots level interventions and engagement in these areas.^78^ Community-driven approaches ensure interventions are tailored to the specific needs and challenges of the community, potentially leading to more sustainable outcomes.

## Discussion

Despite progress made towards the HHSDGs, the pace raises concerns about whether the 2030 target will be met. Examining progress, challenges and opportunities for implementation of HHSDGs revealed advancements; however, recurring themes emerged across studies that highlighted existing challenges with implementing the HHSDGs (online supplemental table 5). The impacts of the COVID-19 pandemic have been irrefutable, marked by lockdowns and data coverage gaps, as well as disruption of essential services, including routine immunisation and general access to health services.^46 129 130^ While not solely attributed to the pandemic, high rates of staff turnover, understaffing and stockouts of essential medicine were identified as common challenges to health delivery,^23^,^2552 68 70 107 114 131^ all of which have inevitably hampered progress towards the SDGs. Coupled with other polycrises, the ability of states to reach the SDGs becomes even more challenging, particularly for those affected by political violence, alongside outbreaks and epidemics and a rising cost of living.^132^,^134^ The impact of climate change is also hindering SDG progress, not only on climate action indicators but also on health and health-related impacts.^135^

Inadequate and inconsistent financing, underbudgeting and the high cost of drugs were identified as additional significant barriers to achieving the HHSDGs.^37 38 40 136^ The immaturity of health-financing systems^40^ and limited understanding of health insurance^23^ added an additional layer of complexity. Resource constraints were also prevalent, exemplified by a lack of education bursaries for adolescent girls, leading to dropouts and weak monitoring systems for interventions to end child marriage.^46 137^

Societal norms and practices emphasise the pressing need to address SDG 5. Child welfare and protection measures, especially in the context of practices such as FGM, further stress the need for safeguarding the rights of children and girls. Strong patriarchal tendencies are highly prevalent in regions of SSA, MENA and SA, where men hold power over women, making important lifestyle and health decisions for them.^40 60^

The state of political and legislative landscapes also heavily influenced implementation, with difficulties passing important legislation tied to health and delayed project initiation due to external dependencies.^46^

The absence of systematic data collection, gaps in evidence and lack of relevant and quality data displayed the critical need for robust data systems in monitoring SDG progress effectively.^44 72 115^

There are numerous examples highlighting progress, challenges and opportunities for implementation of HHSDGs (online supplemental tables 5 and 6). However, each target and subtarget varies, as well as each regional and national context. COVID-19 highlighted pre-existing structural weaknesses—underinvestment, slow reform cycles, weak governance and inequity. Most pre-COVID strategies were framed as commitments, guidelines or frameworks, emphasising future implementation plans, as limited impact data was available given only 4 years of the SDG period had passed. During the pandemic, existing resources were diverted to emergency response measures, with community-based local solutions showing the greatest impact. Postpandemic, there is an emphasis on knowledge exchange, multisectoral collaboration, digital communication platforms and workforce protection for sustainable recovery and progress towards HHSDG implementation.

The Global Sustainable Development report (2023) highlighted the need for key transformative shifts across different entry points and sectors, supporting strategic decision-making by different societal actors, and connecting knowledge to decision-making in a more robust and inclusive manner to accelerate progress toward the SDGs.^138^ Massive disruption of global progress in HHSDGs by the pandemic,^139^ global economic downturn, increased debt burden and reductions in development financing and assistance by many high-income countries have further compounded the slowing of progress.^140^

Addressing these challenges for HHSDGs will require cross-sectoral collaboration, collective or shared action across all levels of government and non-governmental actors based on evidence-informed, cost-effective best practices. Knowledge and evidence gathering and targeted implementation strategies are needed to advance the HHSDGs, particularly in LMICs. For instance, climate and environmental challenges could be addressed through the redirection of global finances, including new indicators to track such initiatives.^141^ Adaptation and mitigation policies can also address climate change and health impacts, among many other initiatives, to transition towards *a clean and healthy future*.^141^

### Recommendations

Efforts to attain UHC (SDG Target 3.8) were prevalent across many included studies and are essential for meeting the HHSDGs targets. Recommended strategies for addressing UHC included enhancing primary care services,^24 62 131^ strengthening public health systems,^31^ adopting frameworks and guidance from expert entities such as the WHO^142^ and increasing interministerial cooperation.^18^

Strengthening emergency health responses was also emphasised, partnered with select international NGOs in ‘emergency’ zones. UNFPA and UNICEF are taking steps to diversify their partnerships with new NGOs in these regions to reduce disruptions to service delivery.^46^

While progress has been made to improve the health, well-being and empowerment of adolescent girls, more work remains for this vulnerable and often overlooked group. Evidence from the included studies shows child marriage and GBV (eg, FGM) are still highly prevalent in many countries. Government financing and community participation were recommended strategies to address this issue. The Adolescent Girls Initiative-Kenya used CCTs to enhance girls’ school attendance and participation in knowledge building workshops.^143^ A key takeaway from this programme is the importance of adopting a multisectoral approach, addressing issues such as child marriage across multiple disciplines including health, education and child protection.^143^

### Implications for future research

Key areas for further research to guide SDG implementation include effective, cost-efficient and multisectoral implementation strategies, which include good governance, accountability and monitoring. Accountability is paramount when implementing monitoring and evaluation (M&E) measures to ensure some entity is ultimately held responsible for ensuring positive progress is made. Strengthening (near-to-real-time) data quality and availability is also essential, disaggregated by sex, population group, (health and/or health-related) outcome and location. Focusing on vulnerable groups, such as women, children and adolescents, is imperative to achieving national, regional and global success. This review highlights a need to revisit SDG progress at various intervals to ensure targets are on track. Additionally, further evaluation of intervention effectiveness could also be examined to understand what and how interventions are advancing the HHSDGs.

### Strengths and limitations

This study conducted an exhaustive search of the literature in the past 5 years and included non-peer-reviewed sources; however, certain implementation strategies may not have been captured if approaches were not documented in the literature. Thus, the study design and reliance on secondary sources of data is an inherent limitation. Furthermore, documents in local languages were not included, limiting full geographical representation of the literature.

## Conclusion

This study suggests that while progress and implementation efforts for the HHSDGs have improved, such efforts continue to differ for various countries and regions. Key challenges centre around continuous financial commitments to ensure a focus on the SDGs remains. More attention is needed to strengthen (near-to-real-time) data availability. Greater multidisciplinary and multistakeholder collaboration should continue to expand and shape the SDG agenda and beyond. Innovative and cost-effective solutions that transcend geographical regions are on the rise and should continue to be supported and encouraged to ensure an upward trend in SDG advancement. Areas for future research include examining the complexity and interplay of multiple SDGs, in addition to the impact of factors beyond the SDGs. Re-examining the SDGs and ensuring they capture the depth of issues faced globally is another area to examine, alongside the complexity of contexts and settings. For example, regions hit by climate change, alongside ongoing conflict or turmoil, and frequent outbreaks/epidemics might advance or regress within the SDG targets. Understanding these factors and how they interplay would be key. Effective and cost-efficient cross-regional approaches for project implementation and monitoring would be useful to facilitate greater knowledge sharing and lessons learnt.

## Supplementary material

10.1136/bmjgh-2025-021623 online supplemental file 1

## Data Availability

Data are available upon reasonable request.
