# Supplementary material for: Implementation of health and health-related sustainable development goals: progress, challenges and opportunities—a systematic literature review update
Source: BMJ Glob Health. 2026 Feb 2;11(2):e021623. doi: 10.1136/bmjgh-2025-021623 (PMC12878203; doi:10.1136/bmjgh-2025-021623)
Supplement: online supplemental file 1 [file bmjgh-11-2-s001.docx]

**SUPPLEMENTAL FILE**

**Table of Contents:**

[Supplemental Table 1a/b: Search strategy 2](#_Toc208237748)

[Supplemental Table 1a: Peer reviewed search 2](#_Toc208237749)

[Supplemental Table 1b: Grey literature search 4](#_Toc208237750)

[Supplemental Table 2: PICO 8](#_Toc208237751)

[Supplemental Table 3: PRISMA checklist 9](#_Toc208237752)

[Supplemental Table 4: Study characteristics and key findings with studies grouped by intervention type 12](#_Toc208237753)

[Supplemental Table 5: Challenges reported by included studies to implementing the SDGs pre-COVID, during COVID and post-COVID 88](#_Toc208237754)

[Supplemental Table 6: Strategies reported by included studies for implementing the SDGs pre-COVID, during COVID and post-COVID 94](#_Toc208237755)

[Supplemental Figure 1: SDG publication trends from 2015-2024 using peer-reviewed data sources 104](#_Toc208237756)

[Supplemental Table 7: The 17 UN SDGs and their prevalence across included studies 105](#_Toc208237757)

[Supplemental Figure 2: Number of studies grouped by intervention category 105](#_Toc208237758)

[Supplemental Table 8: Methodology for Figure 4: Availability of information about HHSDG implementation by sub-domain for all included peer-reviewed studies 106](#_Toc208237759)

## Supplemental Table 1a/b: Search strategy

Search Strategy – All sources updated January 2025

### Supplemental Table 1a: Peer reviewed search

| *Peer Reviewed* |
| --- |
| **Medline**   1. Sustainable Development Goal*.mp. 2. SDG*.mp. 3. 1 or 2 4. "exp PHENOTYPE/ or phenotype*.mp. or subdural grid*.mp. or glycemia standard deviation.mp. or peptide*.mp. or exp Amino Acids/ or amino acid*.mp. or diglucoside*.mp. or histone*.mp." 5. 3 not 4 6. "exp Intersectoral collaboration/ or Intersectoral.mp. or Multisecotral.mp. or Cooperation.mp. or Collaboration.mp. or Impl ementation.mp. or Policy.mp. or legislation.mp. or rules.mp. or Regional.mp. or global.mp. or ""whole-of-government"".mp. or ""Joined-up government"".mp. or 7. ""Healthy public policy"".mp. or ""Health-in-all-policy"".mp. or HiAP.mp. or ""Health-in-all-policies"".mp. or Integrat*.mp. [mp=title, abstract, original title, name of substance word, subject heading word, floating sub-heading word, keyword heading word, organism supplementary concept word, protocol supplementary concept word, rare disease supplementary concept word, unique identifier, synonyms]" 8. 5 and 6 9. "limit 7 to ed=20220531-20230322" |
| **Embase**   1. Sustainable Development Goal*.mp. 2. SDG*.mp. 3. 1 or 2 4. "exp PHENOTYPE/ or phenotype*.mp. or subdural grid*.mp. or glycemia standard deviation.mp. or peptide*.mp. or exp Amino Aci ds/ or amino acid*.mp. or diglucoside*.mp. or histone*.mp." 5. 3 not 4 6. "exp Intersectoral collaboration/ or Intersectoral.mp. or Multisecotral.mp. or Cooperation.mp. or Collaboration.mp. or Impl ementation.mp. or Policy.mp. or legislation.mp. or rules.mp. or Regional.mp. or global.mp. or ""whole-of-government"".mp. or ""Joined-up government"".mp. or ""Healthy public policy"".mp. or ""Health-in-all-policy"".mp. or HiAP.mp. or ""Health-in-all-policies"".mp. or Integrat*.mp. [mp=title, abstract, heading word, drug trade name, original title, device manufacturer, drug manufacturer, device trade name, keyword, floating subheading word, candidate term word]" 7. 5 and 6 8. limit 7 to yr="2022 -Current" |
| **Cab Abstracts**   1. "Sustainable Development Goal*.mp. [mp=abstract, title, original title, broad terms, heading words, identifiers, cabicodes]" 2. SDG*.mp. 3. 1 or 2 4. "exp PHENOTYPE/ or phenotype*.mp. or subdural grid*.mp. or glycemia standard deviation.mp. or peptide*.mp. or exp Amino Acids/ or amino acid*.mp. or diglucoside*.mp. or histone*.mp." 5. 3 not 4 6. "exp Intersectoral collaboration/ or Intersectoral.mp. or Multisecotral.mp. or Cooperation.mp. or Collaboration.mp. or Impl ementation.mp. or Policy.mp. or legislation.mp. or rules.mp. or Regional.mp. or global.mp. or ""whole-of-government"".mp. or ""Joined-up government"".mp. or ""Healthy public policy"".mp. or ""Health-in-all-policy"".mp. or HiAP.mp. or ""Health-in-all-policies"".mp. or Integrat*.mp. [mp=title, abstract, heading word, drug trade name, original title, device manufacturer, drug manufacturer, device trade name, keyword, floating subheading word, candidate term word]" 7. 5 and 6 8. 8 limit 7 to (abstracts and full text & cab abstracts fulltext and yr="2022 -Current" |
| CINAL   1. Sustainable Development Goals 2. Limit - Published Date: 20220601-20230331   TX All Text |
| **Cochrane**   1. Sustainable Development Goals 2. limit 1 to Published Date: 20220531-20230322 |
| **Title, abstract, keywords:** |
| **3ie Databases of Impact**   1. Sustainable Development Goals 2. limit 1 to Published Date: 2022-present |
| **WHO regional databases (WHOLIS)**   1. Sustainable Development Goals |

### Supplemental Table 1b: Grey literature search

| Source searched | Database | **Open Grey** |
| --- | --- | --- |
| Main website URL |  | [www.opengrey.eu/](http://www.opengrey.eu/) |
| Resource page |  | [www.opengrey.eu/](http://www.opengrey.eu/) |
| Searched on |  | Jan 12, 2023 |
| Syntax |  |  |
|  | Used for retrieval | sustainable AND development AND goals |
|  | Variants tested | (sustainable development goals OR Sustainable development goal OR SDGs OR SDG) AFTER 2013 |
|  |  | Sustainable development goals OR /SDG(3\|6\|7\|11\|13)?/ |
| Date range | None |  |
| Returns | 43 |  |
| Comments | Need to look at only those published after 2019 | |

| Source searched | Website |  | **UN high level political forum for SDGs** |
| --- | --- | --- | --- |
| Main website URL |  |  | <https://sdgs.un.org> |
| Resource page |  |  | <https://sustainabledevelopment.un.org/topics/science/documents> |
| Searched on |  |  | January 12^th^, 2023 |
| Syntax |  |  |  |
|  | Used for retrieval |  | sustainable AND development AND goals /SDG(1\|2\|3\|5\|6) |
|  | Variants tested |  |  |
|  |  |  |  |
| Date range | none |  |  |
| Returns                      121 |  |  |  |
| Comments |  | | |

| Source searched | Website | **Google Scholar** |
| --- | --- | --- |
| URL |  | www.google.com |
| Searched on |  | November 14, 2022 |
| Syntax |  |  |
|  | Used for retrieval | (Sustainable Development Goal or SDG or Sustainable Development Goals) AND (Intersectoral or Multisecotral or Cooperation or Collaboration or Implementation or Policy or legislation or Regional or global or Integration or "Action plan") |
|  | Variants tested | None |
|  |  |  |
| Date range |  | 2019 - 2022 |
| Returns | 520 |  |
| Comments | Need to look at only those published after 2019 | |

| Source searched | Website | **WHOLIS** |
| --- | --- | --- |
| URL |  | <http://kohahq.searo.who.int/> |
| Resource page |  | <http://kohahq.searo.who.int/cgi-bin/koha/opac-search.pl?expanded_options=1&returntosearch=1> |
| Searched on |  | Nov 3, 2022 |
| Syntax |  |  |
|  | Used for retrieval | Sustainab$ AND Develop$ AND Goal$ |
|  | Variants tested | Sustainab$ AND Develop$ AND Goal$ AND Health (15) |
|  |  |  |
| Date range |  | None |
| Returns | 18 |  |
| Comments | Need to look at only those published after 2019 | |

| Source searched | Website | **UNDP** |
| --- | --- | --- |
| URL |  | <http://www.undp.org/content/undp/en/home.html> |
| Resource page |  | <http://www.undp.org/content/undp/en/home/library.html> |
| Searched on |  | Jan 12th, 2023 |
| Syntax |  |  |
|  | Used for retrieval | (Sustainable Development Goals) |
|  | Variants tested | Sustainable Development Goals |
|  |  |  |
| Date range |  | None |
| Returns | 146 |  |
| Comments | Need to look at only those published after 2019 (SDG; 1,2,3,5,6) | |

| Source searched | Website | **UNFPA** |
| --- | --- | --- |
| URL |  | [https://www.unfpa.org](https://www.unfpa.org/) |
| Resource page |  | <https://www.unfpa.org/publications> |
| Searched on |  | January 12th, 2022 |
| Syntax |  |  |
|  | Used for retrieval | Sustainable Development Goals |
|  | Variants tested | Sustainab* AND Develop* AND Goal* |
|  |  |  |
| Date range |  | None |
| Returns | 717 |  |
| Comments | 2019 – present; I looked through the results | |

| Source searched | Website | **UNICEF** |
| --- | --- | --- |
| URL |  | <https://www.unicef.org/> |
| Resource page |  | <https://www.unicef.org/search/search.php?querystring_en=%22sustainable+development+goals%22+AND+health+AND+implementation&hits=&type=&navigation=&Go.x=0&Go.y=0> |
| Searched on |  | January 12th, 2023 |
| Syntax |  |  |
|  | Used for retrieval | sustainable development goals AND health AND implementation |
|  | Variants tested |  |
|  |  |  |
| Date range |  | 01/08/2019 to 12/01/2023 |
| Returns | 999 |  |
| Comments | Filtered for Annual report, article, document, flagship report, programme, report, situation report | |

| Source searched | Website | **World** **Bank** |
| --- | --- | --- |
| URL |  | <https://www.worldbank.org/> |
| Resource page |  | <https://openknowledge.worldbank.org/> |
| Searched on |  | January 12th, 2023 |
| Syntax |  |  |
|  | Used for retrieval | "Sustainable development goals" |
|  | Variants tested |  |
|  |  |  |
| Date range |  | 2019(61), 2020(1), 2021(2), 2022(1), 2023(0) |
| Returns | 65 |  |
| Comments | Need to look at journals those published after July 2019 | |

| Source searched | Website | **Institute of Developmental Studies** |
| --- | --- | --- |
| URL |  | <http://www.ids.ac.uk/> |
| Resource page |  | <http://www.ids.ac.uk/publications> |
| Searched on |  | November 3, 2022 |
| Syntax |  |  |
|  | Used for retrieval | Sustainable development goal |
|  | Variants tested |  |
|  |  |  |
| Date range | 2019 to 2023 (no way to select specific dates) |  |
| Returns | 225 |  |
| Comments |  | |

| Source searched | Journal | **IDS Bulletin** |
| --- | --- | --- |
| URL |  | <http://bulletin.ids.ac.uk/idsbo> |
| Resource page |  | <http://bulletin.ids.ac.uk/idsbo/search/search> |
| Searched on |  | November 3, 2022 |
| Syntax |  |  |
|  | Used for retrieval | Sustainable Development Goals |
|  | Variants tested |  |
|  |  |  |
| Date range |  | August 1, 2019 to January 12 |
| Returns | 49 |  |
| Comments | Need to look at only those published after 2019 | |

## Supplemental Table 2: PICO

| PICO |
| --- |
| The same PICO - (Population, Intervention, Comparison and Outcomes) - elements around terms searched were used as that in the previous review. P being ‘health and health-related sustainable development goals’, I being ‘implementation strategies’, and C and O components not having any specifications. The same strategy was utilized around the following terms with some amendments for each database, where needed: ‘Sustainable Development Goal’, ‘Multisectoral’, ‘Collaboration’, ‘Implementation’, ‘Policy’, ‘legislation’, ‘whole-of-government’, ‘Integration’, ‘Plan of action’. |

## Supplemental Table 3: PRISMA checklist

| **Section and Topic** | **Item #** | **Checklist item** | **Location where item is reported** |
| --- | --- | --- | --- |
| **TITLE** | | |  |
| Title | 1 | Identify the report as a systematic review. | Page 1. |
| **ABSTRACT** | | |  |
| Abstract | 2 | See the PRISMA 2020 for Abstracts checklist. | Page 2. |
| **INTRODUCTION** | | |  |
| Rationale | 3 | Describe the rationale for the review in the context of existing knowledge. | Page 2-3. |
| Objectives | 4 | Provide an explicit statement of the objective(s) or question(s) the review addresses. | Page 3. |
| **METHODS** | | |  |
| Eligibility criteria | 5 | Specify the inclusion and exclusion criteria for the review and how studies were grouped for the syntheses. | Page 5 |
| Information sources | 6 | Specify all databases, registers, websites, organisations, reference lists and other sources searched or consulted to identify studies. Specify the date when each source was last searched or consulted. | Page 4. |
| Search strategy | 7 | Present the full search strategies for all databases, registers and websites, including any filters and limits used. | Supplementary file (Supplementary Tables 1a/b.) |
| Selection process | 8 | Specify the methods used to decide whether a study met the inclusion criteria of the review, including how many reviewers screened each record and each report retrieved, whether they worked independently, and if applicable, details of automation tools used in the process. | Page 4-5. |
| Data collection process | 9 | Specify the methods used to collect data from reports, including how many reviewers collected data from each report, whether they worked independently, any processes for obtaining or confirming data from study investigators, and if applicable, details of automation tools used in the process. | Page 5-6. |
| Data items | 10a | List and define all outcomes for which data were sought. Specify whether all results that were compatible with each outcome domain in each study were sought (e.g. for all measures, time points, analyses), and if not, the methods used to decide which results to collect. | Page 4-5. |
|  | 10b | List and define all other variables for which data were sought (e.g. participant and intervention characteristics, funding sources). Describe any assumptions made about any missing or unclear information. | Page 5-6 & Supplement 4. |
| Study risk of bias assessment | 11 | Specify the methods used to assess risk of bias in the included studies, including details of the tool(s) used, how many reviewers assessed each study and whether they worked independently, and if applicable, details of automation tools used in the process. | Page 6. |
| Effect measures | 12 | Specify for each outcome the effect measure(s) (e.g. risk ratio, mean difference) used in the synthesis or presentation of results. | N/A |
| Synthesis methods | 13a | Describe the processes used to decide which studies were eligible for each synthesis (e.g. tabulating the study intervention characteristics and comparing against the planned groups for each synthesis (item #5)). | Page 6. |
|  | 13b | Describe any methods required to prepare the data for presentation or synthesis, such as handling of missing summary statistics, or data conversions. | Page 6. |
|  | 13c | Describe any methods used to tabulate or visually display results of individual studies and syntheses. | Page 6. |
|  | 13d | Describe any methods used to synthesize results and provide a rationale for the choice(s). If meta-analysis was performed, describe the model(s), method(s) to identify the presence and extent of statistical heterogeneity, and software package(s) used. | Page 6. |
|  | 13e | Describe any methods used to explore possible causes of heterogeneity among study results (e.g. subgroup analysis, meta-regression). | N/A |
|  | 13f | Describe any sensitivity analyses conducted to assess robustness of the synthesized results. | N/A |
| Reporting bias assessment | 14 | Describe any methods used to assess risk of bias due to missing results in a synthesis (arising from reporting biases). | N/A |
| Certainty assessment | 15 | Describe any methods used to assess certainty (or confidence) in the body of evidence for an outcome. | N/A |
| **RESULTS** | | |  |
| Study selection | 16a | Describe the results of the search and selection process, from the number of records identified in the search to the number of studies included in the review, ideally using a flow diagram. | Page 6-7. |
|  | 16b | Cite studies that might appear to meet the inclusion criteria, but which were excluded, and explain why they were excluded. | N/A |
| Study characteristics | 17 | Cite each included study and present its characteristics. | Supplement 4 |
| Risk of bias in studies | 18 | Present assessments of risk of bias for each included study. | N/A |
| Results of individual studies | 19 | For all outcomes, present, for each study: (a) summary statistics for each group (where appropriate) and (b) an effect estimate and its precision (e.g. confidence/credible interval), ideally using structured tables or plots. | Page 6-18 &  Supplement 4 |
| Results of syntheses | 20a | For each synthesis, briefly summarise the characteristics and risk of bias among contributing studies. | Page 7-8 & Supplement 4 |
|  | 20b | Present results of all statistical syntheses conducted. If meta-analysis was done, present for each the summary estimate and its precision (e.g. confidence/credible interval) and measures of statistical heterogeneity. If comparing groups, describe the direction of the effect. | N/A |
|  | 20c | Present results of all investigations of possible causes of heterogeneity among study results. | N/A |
|  | 20d | Present results of all sensitivity analyses conducted to assess the robustness of the synthesized results. | N/A |
| Reporting biases | 21 | Present assessments of risk of bias due to missing results (arising from reporting biases) for each synthesis assessed. | N/A |
| Certainty of evidence | 22 | Present assessments of certainty (or confidence) in the body of evidence for each outcome assessed. | N/A |
| **DISCUSSION** | | |  |
| Discussion | 23a | Provide a general interpretation of the results in the context of other evidence. | Page 19-20. |
|  | 23b | Discuss any limitations of the evidence included in the review. | N/A |
|  | 23c | Discuss any limitations of the review processes used. | Page 5. |
|  | 23d | Discuss implications of the results for practice, policy, and future research. | Page 3, 19-20. |
| **OTHER INFORMATION** | | |  |
| Registration and protocol | 24a | Provide registration information for the review, including register name and registration number, or state that the review was not registered. | Register name: Implementation of health and health-related sustainable development goals: progress, challenges and opportunities – a systematic literature review update  Registration number & DOI: <https://doi.org/10.17605/OSF.IO/GNDFY> |
|  | 24b | Indicate where the review protocol can be accessed, or state that a protocol was not prepared. | Protocol can be accessed on Open Science Framework. |
|  | 24c | Describe and explain any amendments to information provided at registration or in the protocol. | N/A, no amendments made to protocol. |
| Support | 25 | Describe sources of financial or non-financial support for the review, and the role of the funders or sponsors in the review. | Page 21. |
| Competing interests | 26 | Declare any competing interests of review authors. | Page 21. |
| Availability of data, code and other materials | 27 | Report which of the following are publicly available and where they can be found: template data collection forms; data extracted from included studies; data used for all analyses; analytic code; any other materials used in the review. | Data synthesis will be available to readers through the supplementary file. Additional material such as template data collection forms, can be shared with specific individuals upon reasonable request. |

*From:*  Page MJ, McKenzie JE, Bossuyt PM, Boutron I, Hoffmann TC, Mulrow CD, et al. The PRISMA 2020 statement: an updated guideline for reporting systematic reviews. BMJ 2021;372:n71. doi: 10.1136/bmj.n71

## Supplemental Table 4: Study characteristics and key findings with studies grouped by intervention type

| **Author, Year** | **Country(ies)** | **Level** | **Study Design** | **SDGs** | **Interventions/ Context** | **Key Findings** |
| --- | --- | --- | --- | --- | --- | --- |
| **Original Search = 30 studies** | | | | | | |
| Abawollo et al, 2022 | Ethiopia | Facility-level | Questionnaire | 3 (3.1) | **Digital health initiatives** | - A portable obstetric ultrasound service provided by trained midwives at the primary health center has contributed to the prevention of 1,970 maternal and 19.05 neonatal morbidities and mortalities per 100,000 and 1,000 live births respectively - Recommendations include scaling up in similar setups to achieve SDG goals 3.2 and 3.4 |
| Amerzadeh et al, 2020 | Iran | National | Policy synthesis | 3 (3.4) | **NCD promotions** | - Iran presents a proactive approach to prioritize NCDs at the highest level and functioning interventions at the lowest level to reach 30% death reduction attributable to NCDs by 2030. - Iran’s pathway, as a WHO fast track country, will help other countries to scale up their active efforts to reach SDG 3.4 |
| Ara et al, 2022 | Bangladesh | Facility-level | Quasi-experimental | 3 (3.3, 3.8) | **WASH initiatives – Waste management** | - A community-led total sanitation approach saw significant improvements in waste segregation practices using color-coded bins, the use of personal protective equipment during transportation and final management/disposal and compliance with the use of standardized methods for collecting and transporting biomedical waste (BMW) |
| Bassoumah et al, 2021 | Ghana | Community-level | Observational | NS | **Community-based health programmes** | - The participants reported poor clinical attendance including delays in seeking health care, low antenatal and postnatal care visits. - In seeking health care, insured members of the national health insurance scheme (NHIS) still pay for services that are covered by the NHIS. Poor community engagement, poor coordination of interventions, poor communication networks during emergencies, and inaccessibility of ambulance service were other barriers. - CHPS compounds also lack the capacity to sterilize some of their equipment, lack of incentives for Community Health Officers and Community Health Volunteers and inadequate infrastructures such as potable water and electricity. |
| Berhan et al, 2022 | Ethiopia | National | Literature review | 3 (3.8),5 | **Sexual and reproductive health programmes** | - Including the SRHR strategy in UHC has helped improve maternal and child health through ANC use and a small decline in child marriages and Female Genital Mutilation (FGM). However, there is still a high prevalence of HIV/AID |
| Borgen et al, 2021 | Democratic Republic of Congo | Institutional-Level | Qualitative | 3 | **Capacity building initiatives** | - Overall, the teaching environment was insufficient. Most midwifery educators and clinical preceptors had deficient competencies, and there was a shortage of didactic resources and equipment as well as poor communication routines between the education institutions and clinical education sites. - Reforming the education of midwives, together with general higher education reform, will be critical for achieving the SDG-3 in the country. |
| Chakrabarti et al, 2021 | India | National | Cross-sectional surveys | 2 (2.2), 3 | **Cash transfer programmes** | - Receipt of the *Mamata Conditional Cash Transfer* was conditional on PLW accessing health services and was associated with increased utilization of maternal health care services, improved maternal nutrition, and reduced food insecurity among treated households. |
| Chhajed-Picha et al, 2021 | India | Subnational | Cross-sectional study | 6 | **WASH initiatives - Waste management** | - Decentralization reforms of the solid waste management system - A long-term collaboration between local colleges and local government or public utility could be a potential gamechanger for various endeavors in development planning and cooperation, and urban planning. - SFD gives the impression that the majority (68%) of OSS safely contain excreta. |
| Choorakuttil et al, 2022 | India | Facility-level | Cohort study | 3 (3.2) | **Capacity building initiatives** | - Fetal Doppler integrated antenatal ultrasound studies in *Samrakshan* led to a significant reduction in preterm pre-eclampsia rates, preterm birth rates, and a significant improvement in mean birth weights. Perinatal, neonatal, and maternal mortality rates are significantly better than the targets for 2030 set by SDG-3 |
| Echaubard et al, 2020 | Cambodia | Community-level | Program evaluation | 3 (3.8) | **Community-based health programmes** | - In Cambodia, there is a need for simple, low-cost solutions to improve health care and to heal the fragmentation between policymakers and ground-level solutions. - The initiative described in this article put in motion processes of community engagement towards creating ownership of dengue control interventions tools by community stakeholders, including school children. |
| Ekenna et al, 2020 | Nigeria | Facility-level | Cross-sectional study | 3 (3.8) | **Community-based health programmes** | - An integrated system linking primary health centers (PHCs) and secondary care facilities in Enugu State, still found significant gaps that need to be closed for PHC to make significant contributions towards achieving universal healthcare, core to achieving SDG-3 - None of the PHC centers surveyed had all the recommended service domains, but 87% offered at least half of the recommended service domains |
| Gonzalez-Rodrigo et al, 2022 | Tanzania | House-hold-level | Comparative analysis | 6 (6.2) | **WASH initiatives** | - The implementation of the Community Led Total Sanitation approach to stop Open Defecation (OD) programme led to a decrease of OD to 9.70% and achieving several OD free villages, increase in constructed latrines and increase in access to sanitation facilities like hand washing in communities who were far from meeting target 6.2 of the 2030 SDGs |
| Hands et al, 2021 | Sierra Leone | Regional-level | Retrospective analysis | 3 (3.1, 3.2) | **Health insurance schemes** | - The Free Health Care initiative for children under five, pregnant women, and lactating mothers led to an increase in hospital admission rates, a reduction in hospital wait time, increased quality of care for children with respiratory diseases, a reduction in child mortality |
| Kongmany et al, 2020 | Lao PDR | Facility-level | Cross-sectional study (Descriptive) | 3 (3.8) | **Health insurance schemes** | - Integration of existing social health protection schemes into a single payer system was successful in reducing out of pocket payment and providing substantial coverage for various medical services |
| Kumar et al, 2021 | India | Household-level, facility-level | Retrospective study | NS | **Capacity building initiatives** | - Implementation of the Integrated Management of Neonatal and Childhood Illness (IMNCI) package saw Improvements in under-nutrition and wasting |
| Lindberg et al, 2021 | Uganda | Facility-level | Cross- sectional study | 3 (3.1, 3.8) | **Health insurance schemes** | - *Ugandan National Minimum Health Care Package* - Majority of the women (91%) paid for their delivery due to lack of health insurance coverage - Service coverage was highest for Skilled Birth Attendants and institutional delivery (88%) and lowest for a postnatal visit (54%). Forty percent of women delivered in hospitals and a third in health centers (33%). Most women (68%) reported at least four ANC visits. |
| Meilani et al, 2019 | Indonesia | Facility-level | Cross-sectional study | 3 (3.3) | **Capacity building initiatives** | - The implementation of the *PMTCT Program* to build capacity of midwives to encourage HIV testing for pregnant women has not been successful as it has only been 52.5% effective. |
| Mohd Hanafiah et al, | Malaysia | Facility-level | Descriptive study/Implementation research | 3 (3.8) | **Community-based health programmes** | - The *"one doctor one family"* policy through the FHT program was adhered to and has created familiarity between patients and their healthcare providers. - Patients are now more willing to share information pertaining to their health and are more consistent with attending hospital appointments. Quality of care was also improved due to personalization. - In places where familiarity was not attained, it discouraged patients from seeking care. |
| Nadella et al, 2021 | India | National | Cross-sectional study | 3 (3.1, 3.2) | **Community-based health programmes** | - Receiving care from ASHAs and AWWs were each independently associated with improved ANC utilization and quality as well as early initiation of breast feeding. Only receiving ANC from ASHAs was independently associated with decreased one-year mortality. |
| Ndiaye et al, 2021 | Senegal | Community-level & Household-level | One-group pretest-posttest design ( repeated cross-sectional design) | 3 (3.1) | **Community-based health programmes** | - *Better Maternal Health*– information, education and communication (IEC) campaigns, home visits and talks with the villagers |
| Odagiri et al, 2020 | Indonesia | District-level | Cross sectional study (large-scale effectiveness evaluation) | 6 | **WASH initiatives - Waste management** | - *Community-led total sanitation approach to develop Sanitation and Hygiene acceleration plans* - High intensity districts were more likely to be ODF (aRR 4.65, CI 2.12–10.20) with greater increase in household toilet coverage (aRR 11.15 CI 1.04–119.82). Weaker non-significant associations with ODF were observed among learning districts relative to comparison districts. |
| Otieno et al, 2022 | Ghana | Household-level | Quasi-experimental, longitudinal impact evaluation | 3 (3.8) | **Cash transfer programmes** | - In the quest for expanding financial protection towards accelerating the achievement of universal health coverage, policymakers in Ghana should prioritize the integration of efforts to simultaneously address demand- and supply-side factors. - The study concluded that high service quality amplified the impacts of *Livelihood Empowerment Against Poverty (LEAP) 1000* on NHIS enrolment among all groups examined. |
| Prakash et al, 2020 | India | National | Program evaluation | 3 (3.8) | **Health insurance schemes** | - *Ayushman Bharat -* A need-based health care scheme that provides for hospitalization cover in respect of secondary and   tertiary care up to INR 500,000 for the family   - The scheme is reasonably successful in providing financial relief but need to look at areas concerning quality delivery to achieve UHC goals. |
| Schwarz et al, 2020 | Nepal | National | Longitudinal study (Policy and program analysis) | 3 (3.1, 3.8) | **Community-based health programmes** | - *FCHV program* – The Female Community Health Volunteers provide services including family planning, oral rehydration salts, vitamin A distribution, and outreach clinics. - Nepal’s progress towards the SDGs is commendable, yet questions about whether the design and capacity of the FCHV program can continue to advance Nepal on its trajectory towards SDG target |
| Stansert et al, 2020 | South Africa | Facility-level & Household-level | Longitudinal prospective observational cohort study | NS | **Community-based health programmes** | - *CHW home visits* resulted in better maternal caretaking but did not have direct benefits for infants in the domains assessed. |
| Toure et al, 2021 | Mali | National | Case study | 3 (3.8) | **Capacity building initiatives** | - To introduce *WAHO’s regionally accepted, competency-based nursing curriculum in reproductive, maternal, newborn, and child health* in private schools - Eight schools in Mali are now accredited compared to only three at project inception |
| Utomo et al, 2021 | Indonesia | Facility-level & National | Decomposition study | 3 (3.1) | **Sexual and reproductive health programmes** | - Substantial reductions in maternal mortality between the years 1970 and 2017 can be attributed to the success of the *National Family Planning Program*, and there are prospects for further contributions to the year 2030. - However, the ability of Indonesia to reach the 2030 SDG maternal mortality target will depend more heavily upon the effectiveness of the health system in addressing health risks to women once they are pregnant. |
| Var et al, 2020 | Cambodia | Facility-level & Household-level | Randomized control trial (RCT) | 3 (3.2) | **Community-based health programmes** | - The intervention included health center staff training and home visits to mothers by *community health volunteers* within 24 h of birth and on days 3 and 7 after delivery, including assessment of newborns for danger signs and counselling mothers. - The intervention improved knowledge and self-reported behavior of health staff and health volunteers, as well as mothers’ own knowledge of newborn danger signs but had no impacts on health seeking behavior of the mothers |
| Vonk et al, 2021 | Zambia | Household-level and Community-level | Quasi-experimental Evaluation study | 6 | **WASH initiatives** | - Overall, the *Urban WASH* project did not have a significant impact on the Sustainable Water and Sanitation Index overall (although several significant impacts are observed among the dimensions and indicators, and there is a significant positive impact among households having members with a disability). The project had a significant negative impact on Water Insecurity (HWISE; 2.03, p<0.05). |
| Zhao et al, 2020 | China | National & Subnational | National longitudinal study | 3 (3.2) | **Health insurance schemes** | - *Basic Public Health Service (BPHS) project:* There were statistically significant immediate effects in improving MMRs and neonatal visit rates. Statistically significant long-term effects on these neonatal related services were also shown, since it kept a decreasing trend before the introduction of the project - China had achieved the target to further reduce NMR and to narrow the gap of NMR between urban and rural areas partly through the implementation of the BPHS project. |
| **Updated search (March 2023) = 16 studies** | | | | | | |
| Bai et al, 2022 | China | National | Policy analysis | 2, 3, 6 | **Urban planning** | - The *Healthy Cities initiative* in China has the potential to provide the framework for intersectoral governance and joined-up program planning and implementation to achieve SDGs and Healthy China 2030 at the local level |
| Bryce et al, 2021 | Cambodia | Facility-level | Mixed methods (Qualitative + Quantitative) | 1, 3, 4 8, 10, 17 | **Capacity building initiatives** | - *Partnerships* with key government ministries and universities (SDG 17) contribute to the fulfilment of SDGs by reducing poverty (SDG 1) and inequalities (SDG 10) experienced by Cambodians with communication and/or swallowing disability and strengthening both health (SDG 3) and education systems (SDG 4) with the aim of a future robust, well-defined SLP workforce (SDG 8). |
| Dada et al, 2022 | South Africa | National | Report | 2, 3 (3.4, 3.8), 4 | **Digital health initiatives** | - The work of the *Centre for Augmentative and Alternative Communication (CAAC)* spans various SDGs (SDG 2, SDG 3, SDG 4, SDG 8, and SGD 16) to move beyond impairment-focused interventions. |
| Maponga et al, 2022 | Zimbabwe | National | Report | 3 (3.8) | **Capacity building initiatives** | - *Good Governance for Medicine* – improve supply of medicines 1) strengthening academic public-private partnerships, 2) Incorporation of GGM into pharmacy curricula and motivation for the establishment of district drug information centres, 3) Linkages with emerging priorities in the therapeutic and policy environment, 4) Creation and establishment of the Parliamentary Pharmaceutical Caucus. |
| Nuri et al, 2022 | Bangladesh | National | Policy analysis | 10 | **Health equity programmes** | - The results affirm the government’s effort toward Sustainable Development Goals in providing support to children with disabilities (CWDs) and their families. - The findings highlight the need to embrace the concepts of autonomy, confidentiality, and family-centeredness in great detail in any policy initiatives pertaining to CWDs. |
| Goc et al, 2022 | Sudan | Institutional-Level | Mixed-method evaluation | N/A | **Capacity building initiatives** | - *The* *Yale–Sudan Program* *for Research Leadership in Public Health* achieved high levels of participant engagement (93% graduation rate; 100% would “definitely recommend” to their peers); significant impact on mentorship and leadership competencies; and measurable strengthening of research collaborations within and across partner institutions but sustainability of achievements is yet to be determined |
| Harvey et al, 2022 | Niger and Nigeria | National | Descriptive study | 10 | **Health equity programmes** | - *The Ascend programme* has helped contribute to addressing issues of coverage and inclusion for IDPs and refugees - Without a commitment to and action on “Leave No one Behind”, NTD elimination agendas may fall short of succeeding. |
| Mariwah et al, 2022 | Ghana | National-level, community-level | Sequential mixed-methods approach | 3 (3.8) | **Digital health initiatives** | - *MOTECH programme (Mobile Technology for Community* *Health)* - When properly implemented and regulated, ‘informal’ mHealth will contribute to the achievement of universal health coverage by bridging the healthcare gap between the rich and the poor as well as between rural and urban areas |
| Sibuyi et al, 2022 | South Africa | Facility-level, community-level, organizational-level | Ethnographic approach | 3 | **Digital health initiatives** | - Experience has shown that the scalability and sustainability of mHealth services as part of an innovative Digital health initiatives ecosystem could be hamstrung by factors such as stakeholder mismanagement, lack of political support, appropriate choice of technology, funding, and integration of mHealth into existing health programs in tandem with the SDGs |
| Sowden et al, 2023 | Uganda, Kenya, and Rwanda | National | Commentary | 3, 4, 8, 9, 10, 17 | **Capacity building initiatives** | - *Speech-language pathology services* for people with communication disabilities in East African countries, including Uganda, Kenya, and Rwanda, have often developed through partnerships with Minority World countries, including the UK. - Partnerships for the goals (SDG 17) have worked towards supporting good health and well-being (SDG 3), quality education (SDG 4), decent work and economic growth (SDG 8), industry, innovation and infrastructure (SDG 9) and reduced inequalities (SDG 10). |
| Gao et al, 2022 | China | Provincial level | Longitudinal study design | 3, 10 | **Urban Planning** | - The *Belt and Road Initiative* has significantly improved the residents’ health along the route, while the Pilot Free Trade Zone, which is another important opening-up policy in China, has had an inhibitory effect on the health of residents. - This study will support and advance the UN’s Sustainable Development Goals (SDGs), especially SDG3 (Good Health and Well-being) and SDG10 (Reduced Inequalities). |
| Jacobs et al, 2023 | Zambia | Household-level | Mixed methods (Qualitative + Quantitative) | 3 (3.2) | **Capacity building initiatives** | - Zambia achieved major improvements in reducing inequalities for under-five mortality and coverage of RMNCH as well as malaria and HIV prevention interventions which largely stem from intentional *health sector changes* in a wide range of policies, strategies and investments at the community and facility levels that centrally valued equity. |
| Mabe et al, 2022 | Ghana | Household-level | Secondary Data analysis | 1 | **Cash transfer programmes** | - Currently, African countries have shown their commitment to achieving most SDGs by implementing various kinds of social protection programmes, and Ghana is not an exception - Specifically the *Livelihood Empowerment Against Poverty (LEAP*), which aims to alleviate short-term poverty and encourage long-term human capital development |
| Nugroho et al, 2022 | Indonesia | National | Literature review | 1, 2, 3,  4, 5 | **WASH initiatives** | - A decentralized system can effectively achieve national goals if each local government has a consistent structure and implementation strategy. |
| Odendaal et al, 2022 | South Africa | Facility-level | Case study/Program evaluation | 3 (3.1) | **Capacity building initiatives** | - *Quality improvement initiative for maternal, newborn and child health* - Despite a substantial decline in the MMR, much work still needs to be done to achieve the Sustainable Development Goal for an MMR of less than 70/100,000 by 2030 |
| Puigvert et al, 2022 | Spain | National | Program analysis | 5 | **Sexual and reproductive health programmes - GBV** | - *ProWomenDialogue* – an action platform against violence social impact, aligned with SDG 5, inspires the reduction of GBV, while encouraging the career promotion of female researchers. |
| **Grey literature = 95 studies** | | | | | | |
| WHO 2021 | Fiji | National | Report | 3 (3.4) | **Product regulations - two types of pesticides (paraquat and imidacloprid)** | - The *ban on the importation, sale and use of the two pesticides* was successful in reducing the rates of suicide as data from 2020 suggested a slight decrease in suicide rates when compared to 2019. |
| Mondal 2021 | India | State-level | Policy Analysis | NS | **Product regulations - tobacco** | - Multisectoral policies and practices for *tobacco control* need to be supported by a nurturing multi-level (national and state) policy environment that provides adequate decision space and leadership at the local level, leaving space for experimentation and adaptation in line with local conditions. |
| Khushitor et al, 2022 | South Africa | National | Qualitative | 2 | **Nutrition and food security** | - After years of implementation, these policies have demonstrated some impacts on improving stunting, but not over-nutrition, micronutrient deficiency and environmental security. Overall, most government initiatives emphasize agricultural productivity, and not the root causes of food insecurity, including structural poverty, |
| Abubakar,  Nitzan, Alp Meşe et al, 2019 | Syria | National | Book | 1 (1.3), 3, 10 (10.7) | **Capacity building initiatives** | - In order to strengthen the capacity of Syria and Turkey with helping improve refugee health outcomes, the WHO has been successful in building collaboration between two countries by providing support in the areas of greatest need, such as the training of a *Syrian health workforce* |
|  | Turkey | National | Book | NS |  | - The training and certification of the *Syrian health workforce* was a challenge since the legal framework in Turkey did not allow refugee health professionals to work in national settings and new legislative regulations had to be adopted. |
|  | Turkey | National | Book | 3 (3.1, 3.2, 3.3, 3.4) |  | - Turkmenistan has adopted the *Global Action Plan* and has developed ways of implementing HrSDGs in the country through guided steps provided by the GAP |
| Shamurudova et al, 2020 | Turkmenistan | National | Report on MICS survey | 3 (3.1-3.8) | **Health equity programmes** | - According to the Sustainable Development Report, Turkmenistan is on track to achieve SDG 1, SDG 6, SDG 7 and SDG 8; SDG 3 and SDG 7 have improved but progress is insufficient to achieve these goals by 2030; SDG 2, SDG 5, SDG 9, SDG 11 and SDG 15 are moving very slowly, with progress of less than 50% and possible stagnation on some goals. |
| Habicht et al 2020 | Ukraine | National | Mixed methods – literature review and interviews | 3 | **Health equity programmes** | - As a result of SDG implementation, HIV/AIDS testing, screenings and antiviral therapy has increased. - Secondly, implementation of programs like The National Action Plan on Non-communicable Diseases Prevention, Control and Health Promotion, which was adopted in 2018, has helped reduce the incidence of NCDs. - Thirdly, out of pocket payments have reduced due to government's reform on *health system financing by covering the state guaranteed package of services*, which includes primary medical care, emergency medical care, secondary care (inpatient and outpatient), rehabilitation, palliative care and diagnostics; this has contributed to Ukraine's move towards UHC. |
| Ahart et al, 2020 | Kyrgyz | National | Report | 3 | **Health equity programmes** | - According to the latest Sustainable Development Report, the Kyrgyz Republic occupies 48th place among 162 countries. Specific to SDG 3 (health and well-being), the country is assessed as having achieved close to 75% of its targets which is considered “moderately good progress” towards achieving the SDGs for health. |
| WHO 2020 | European region but with specific country results | National | Report | 1, 2, 3, 4, 5, 10 | **Nutrition and food security** | - *Baby-Friendly Hospital Initiative* to support infant and young child feeding - The report detailed that the impacts of the initiative in some countries has led to the increase in breast feeding rates, notably in Tajikistan and Turkmenistan. - As a region, Europe were well off track to achieve the commonly agreed goals and acknowledged that attaining them will require renewed effort and commitment. |
| Whiting et al, 2019 | North Macedonia | National | Report | 3 (3.4) | **NCD promotions** | - There is strong multisectoral work that is moving forward the plans for implanting policies regarding *physical activity* in North Macedonia. - However, there are certain gaps that need to be addressed to ensure the programmme is beneficial to all populations |
| WHO 2020 | Tajikistan | National | Report | 3 | **Health equity programmes** | - The cross-border nature of the economic, environmental and social problems reinforced the importance of utilizing intersectoral mechanisms, as well as legal instruments, to tackle health-related issues within these domains and deliver on the SDGs. |
| Bai 2022 | China | National | Analysis | 3 | **Urban Planning** | - The *Healthy Cities initiative* in China has made progress on many fronts. More local governments are putting health high on their political agendas, a first step toward "Health in all policies". Quick health improvements were apparent in cities that piloted the initiative. - The Healthy Cities initiative in China has the potential to provide the framework for intersectoral governance and joined-up program planning and implementation to achieve SDGs and Healthy China 2030 at the local level |
| WHO 2021 | Thailand | National | Handbook | NS | **NCD promotions** | - The project has shown the importance of an open data platform to support evidence-based policies for *physical activity* promotion in Thailand. - The short-term success of the platform created opportunities to sustain it beyond the project’s lifecycle. |
| Tatah et al 2021 | Cameroon | National | Analysis | 3 | **NCD promotions** | - The promotion of *physical activity to prevent NCD*s and improve health and wellbeing has progressively been acknowledged, although only the health sector has taken an interest in the health benefits of PA. - The findings of this study point to the need for Cameroonian NCD and PA related policies to work towards adopting a stronger approach for the development of whole of society policies, health in all policies, and intersectoral policies. |
| WHO 2022 | Bahrain | National | Report | 3 (3.6) | **Road safety measures** | - Over the whole study period, the numbers of ambulance calls and minor injuries reduced (by 31% and 29%, respectively), but there were no significant reductions in the numbers of serious injuries and deaths. - As there were no changes in reporting mechanisms during the period of observation, the study suggests a real decline in ambulance calls and minor injuries due to *compliance with the new law.* - The study results also showed a significant reduction in the number of calls for an ambulance for road traffic victims after the law was applied. |
|  | Palestine | National | Report | 3 (3.8) |  | - *The Road Traffic Accidents Information System* - The quality of reported RTA data increased by 38% from 2017 to 2019, and compliance by emergency rooms in hospitals with automatic linkage to the RTA e-registry increased by 40%. - Some 75% of non-governmental health facilities are committed to reporting on RTA, and the quality of reported RTA data increased by 23.4% from 2017 to 2019. |
|  | United Arab Emirates | National | Report | NS |  | - The positive impact of interagency integration on national efforts to reduce deaths, and the benefits of specifying a time period of no more than 100 days to implement *plans to improve traffic safety*, were among the important lessons learned in the course of implementing the accelerator approach. - Modern technologies such as artificial intelligence systems can also help in reducing death rates |
| WHO 2022 | South Sudan | National | Report | 3 (3.8) | **Capacity building initiatives** | - *The health sector stabilization and recovery plan* is still in its early stages and has gained high-level political buy-in and commitment at the national level. - This has been facilitated through continued engagements, coordination and participation of key stakeholders with the resuscitation of the Health Sector Working Group and through the creation of a Health Sector Secretariat with Ministry of Health staff designated as project focal persons. |
| Akoriyea 2019 | Ghana | National | Descriptive Analysis | 3 (3.6) | **Road safety measures** | - Goal 3.6 is relevant to tackling RTA and the Ministry of Health has a major role in implementing it with the aid of its agencies and those from other sectors such as the Ministry of Interior through its Motor Transport and Traffic Department; Ministry of Transport’ Drivers and Vehicle Licensing Authority and National Road safety measures Authority to enforce road traffic regulations and promote Road safety measures; the Ministry of Roads and Highways to improve on road constructions and maintenance; the Ministry of Information’s National Media Commission to engage in active health promotion and preventive activities among others. |
| WHO 2019 | Georgia | National | Report | 2, 3, 4 | **Infectious disease management** | - *The United Nations Common Position on Ending HIV, TB and Viral Hepatitis* in Georgia was successful in helping contribute to the reduction of TB morbidity rates and HIV incidence rates. |
| Olivier et al, 2019 | Malawi | National | Report | NS | **Nutrition and food security** | - The analysis of key aspects of the Malawi domestic *Food Security and Nutrition framewor*k does not provide evidence of adherence to the sequential nature of the policy loop. - In several instances, annual implementation (work) plans are not preceded by sectoral policies, sectoral legislation and/or medium-term sectoral implementation strategies. - In addition, binding constitutional provisions are not, or not fully, embodied in the current Malawi constituent elements of the policy loop. - Furthermore, detailed provisions relating to both an overarching M&E framework (structure and systems) and concomitant customized sub-sector M&E frameworks (structure and systems), are often lacking |
| WHO 2022 | Republic of Kazakhstan | National | Report | 3 | **Health insurance schemes** | - In 2019, 238,100 more people in Kazakhstan (1.2% of the population) were covered by essential health services than in 2018. By 2023, this number is projected to be 886 400 more people (4.6% of the population) - In 2021, the Government of the Republic of Kazakhstan approved the National Project, high-quality and affordable healthcare for every citizen called *Healthy Nation,* for the implementation period 2021–2025. - Coverage of essential health services is defined as the average coverage of the 14 SDG 3.8.1 tracer interventions. Of these 14 indicators, positive progress is projected for nine of them in Kazakhstan by 2023 |
| WHO 2022 | Kyrgyz Republic | National | Report | NS | **Health equity programmes** | - In Kyrgyzstan, the health sector is widely recognized as a leader in terms of aligning national priorities with SDG goals by integrating SDG goals and indicators into Health 2030 and following an intersectoral, whole-of-government approach to policy development and implementation. - Kyrgyzstan has been able to achieve the goal of reducing the global maternal mortality ratio to less than 70 per 100 000 live births. |
| WHO 2019 | India | National | Report | 3 | **Health equity programmes** | - The *Country Cooperation Strategy (CCS)* has led to - strong political momentum to improve the health of India’s population, an explicit government commitment to double public spending on health to 2.5% of GDP by 2025, an increased commitment to move towards universal health coverage by increasing access to free health-care services and by focusing on reaching poor and vulnerable populations |
| Ramani et al, 2020 | Indonesia | National | Review | 1, 5 | **Health equity programmes** | - Although the importance of considering gender, poverty and disability in program design and targeting was mentioned throughout the *Action Plan* there was limited evidence to suggest that programs within the Action Plan are specifically designed to address or assess these issues. Out of the 5 pillars of the National Action Plan, 3 discussed poverty and only one pillar discussed gender and disability. |
| Phulkerd et al, 2021 | Thailand | National | Review | 2 (2.2), 3 (3.4) | **NCD promotions** | - Overall, the implementation of Thai governments covered the majority of the recommended components in each policy area. - The implementation of specific policies did not necessarily result in better outcomes if governments lack appropriate infrastructure in systems to support the implementation of policy. |
| Goel et al, 2022 | India | National | Review | All | **Product regulations - tobacco** | - The *"tobacco endgame*" approach requires collaboration and capacity building of several sectors and stakeholders to align their activities with the tobacco endgame goals and vision of the Government of India. - There is the need for a robust unified approach that engages all the stakeholders and involves increased investment in tobacco control by the country's governments and region. |
| WHO 2021 | Bangladesh, Ethiopia, Ghana, Kenya, Liberia, Malawi, Nigeria, Pakistan, Sierra Leone, South Sudan, Tanzania, Uganda, Zimbabwe | National | Qualitative Report | 1, 2, 3, 4, 5, 16 | **Health equity programmes** | - *Joint external evaluations and national action plans for health security* - National legislation policy and financing – some countries have legal framework and existing policy or administrative instruments, public health workforces and multisectoral coordination mechanisms in place to implement action plans |
| UNHLPF 2020 | Cambodia | National | Comparative case study | NS | **Capacity building initiatives** | - According to a report of the Ministry of Industry and Handicraft (MIH) in 2018, Micro-Small and Medium Enterprises (MSMEs) provided over 70% of jobs and contributed about 58% of the annual Gross Domestic Products (GDP). - However, most Cambodian enterprises are informal without official registration. Out of all enterprises in Cambodia, only 3.5% have registered with the Ministry of Commerce. |
| UNICEF 2022 | Philippines, Paraguay, Jamaica, | National | Technical Guidance | 3 (3.6), 11 (11.2) | **Road safety measures** | - Developing, implementing and maintaining a safe, healthy and sustainable transport system is best attained when actions are integrated across sectors and disciplines. - UNICEF programme areas at global, regional and country offices are well positioned to include Road safety measures activities within their scope of work and across programmes for joint benefits and engagement of partners from diverse sectors. |
| UNICEF 2022 | Kenya, Bangladesh, Ethiopia | National | Report | 5.3 | **Sexual and reproductive health programmes –Child Marriage** | - The programme led to a significantly smaller percentage of girls in the intervention group marrying, as compared to the control group and raised critical awareness skills. - It also led to lower rates of school dropout and analytical learning loss. |
| WHO, UNICEF 2022 | Ethiopia, India, Kenya, Portugal, Uganda, India, Malawi, Kenya | National | Report | 6 (6.1) | **WASH initiatives** | - Highlight the potential of *innovative models of professional maintenance services and accountable and transparent rural water service delivery.* - Between 2015 and 2020, Ethiopia drastically reduced the proportion of its population reliant on surface water and saw an 8% increase in those using at least basic drinking water services - Between 2019 and 2022, India estimated the coverage of functional household taps in rural areas increased from 17% to over 49%. |
| UNICEF 2022 | Bhutan, Cambodia, Jordan Kenya, Rwanda, South Africa, Tanzania, Uzbekistan | National | Report | 4.2 | **ECD** | - Government leadership and ownership of investing in the early years have significantly improved early childhood education indicators in several countries. - In Bhutan, ECE participation rates have increased from 23,4% in 2018 to 31.81% in 2021. - In Jordan, support from the government as well as other donors and stakeholders has resulted in an increase of KG1 and KG2 enrolments in rural and poor areas from 38% in 2009 to approximately 48% in 2021 - Using community centres for ECE is a good mechanism to improve ECCD service delivery. Tanzania initiated satellite centers for far-to-reach children to ensure that even disadvantaged children residing in remote rural and far from schools are enrolled. |
| UNICEF 2021 | Bangladesh, Burkina Faso, Ethiopia, Ghana, India, Mozambique, Nepal, Niger, Sierra Leone, Uganda, Zambia | National | Report | 5.3 | **Sexual and reproductive health programmes –Child Marriage** | - The COVID-19 crisis underscored the vulnerability of adolescent girls in rural and remote areas - Adoption of digital and mass media approaches demonstrated a high potential for sustaining engagement of adolescent girls at scale, although the digital divide should be considered |
| UNICEF 2022 | Tunisia, Afghanistan, Kenya, Egypt, Ethiopia, Philippines | National | Report | 1 | **Health equity programmes** | - In Afghanistan, UNICEF rapidly scaled up its cash programme, reaching 266,151 people by December 2021. This scale-up will continue in 2022 and is expected to reach 1.5 million people by the first quarter of 2022. - In Kenya, UNICEF partnered with the private sector to make workplaces mother and baby friendly. 48 companies have established breastfeeding rooms. |
| UNFPA UNICEF 2021 | Burkina Faso, Djibouti, Egypt, Eritrea, Ethiopia, Gambia, Guinea, Guinea-Bissau, Kenya, Mali, Mauritania, Nigeria, Senegal, Somalia, Sudan, Uganda, Yemen | National | Report | 5 (5.3) | **Sexual and reproductive health programmes – FGM** | - In Djibouti, FGM prevalence has sharply declined in the cohort aged 15-19, from 94.3% in 1994 to 21.1% in 2019. - Since 2018, law enforcement resulted in arrests and convictions for FGM in several countries. |
| UNICEF 2021 | Indonesia, Malaysia, Cambodia, Lao, Myanmar, Philippines | National | Report | NS | **Health equity programmes** | - Good communication, as well as an established network with government authorities and communities of *people with disabilitie*s are assets in gaining the trust of persons with disabilities. - Major drivers for success included the presence of an already existing coordinating body located within the government (i.e., the Subcommittee), its diverse representation from relevant national government agencies and CSOs, and support from the government through the Council for the Welfare of Children. |
| UNICEF Liberia 2021 | Liberia | National | Country Office Annual Report | 3, 5 | **Sexual and reproductive health programmes** | - UNICEF’s response focused on strengthening risk communication and community engagement; promoting vaccine uptake; ensuring continuous access to education; accelerating child protection services; supporting adequate health care, immunization and access to essential nutrition services for children, women and vulnerable communities; supporting hygiene and sanitation promotion; and generating evidence for planning and advocacy. |
| UNICEF Ghana 2021 | Ghana | National | Country Office Annual Report | NS | **Health equity programmes** | - UNICEF Ghana prioritized five key results for children (increasing immunization, nutrition, learning outcomes, and ending violence and open defecation), with a focus on gender equality, equity, inclusion and innovation |
| UNICEF Bolivia 2021 | Bolivia | National | Country Office Annual Report | 6 | **Health equity programmes** | - During the Country Programme’s fourth year, UNICEF positioned child rights, the 2030 agenda and ‘leaving no child behind’ squarely on the agendas of the new authorities and the public while strengthening risk-informed systems and emergency programming using a humanitarian-development nexus approach |
| UNICEF Oman 2021 | Oman | National | Country Office Annual Report | 2, 3, 4, 5 | **Health equity programmes** | - Results achieved ensured better outcomes for children in Oman, in line with UNICEF’s Global Strategic Plan Areas 1, 2, 3 and 5, the Convention on the Rights of the Child, the Convention on the Rights of Persons with Disabilities, and the 2030 Agenda. |
| UNICEF Cameroon 2021 | Cameroon | National | Country Office Annual Report | NS | **Community-based health programmes** | - UNICEF learnt that when local decision-makers access the right information, they understand what is at stake, they come together in networks and influence allocation of domestic resources. - The UNICEF – UNHCR Blueprint initiative enables optimization of interventions in the field through harmonization of strategies and complementary mandates. |
| UNICEF Uzbekistan 2021 | Uzbekistan | National | Country Office Annual Report | 4 (4.2) | **Capacity building initiatives** | - Leveraging Government’s efforts in introducing innovative programmes with UNICEF’s global technical knowledge and support is crucial in enhancing the impact of the innovations and alternative provisions |
| UNICEF Nicaragua 2021 | Nicaragua | National | Country Office Annual Report | 2, 3, 4, 5 | **Health equity programmes** | - Every Child Survives and Thrives (SDG 2,3,4,5): UNICEF supported the Ministry of the Family (MIFAN) to advance the institutionalization of the Child Development Care approach, which has been integrated into the educational curriculum for children aged 0–3 years. - Every Child Learns (SDGs 4 and 5): UNICEF supported the Ministry of Education (MINED) in improving the quality, equity and relevance of education in the three educational levels - Every Child is Protected from Violence and Exploitation (SDG 10 and 16): capacity building of MIFAN to initiate the implementation of the Protocol for Attention to Survivors of Sexual Violence. |
| UNICEF 2022 | Nigeria | National | Report/Case Series | 2 | **Nutrition and food security** | - Together, UNICEF and DSM were able to bring together multisector stakeholders, including Nigeria’s business community under the SUN Movement and its Business Network, to develop a ‘big vision’ and concrete actions to address malnutrition - As of December 2019, just over 120 private sector companies had been successfully enrolled as Network members. |
| UNICEF Uruguay | Uruguay | National | Country Office Annual Report | 3, 4, 13 | **Sexual and reproductive health programmes** | - Adolescents were both greatly affected and had their behavior questioned during the pandemic. UNICEF sought to generate empathy in adults and support for adolescents, contributing to SDGs 3, 4 and 13 - The pandemic increased the visibility of mental health issues in children and adolescents, and these issues will be a central part of UNICEF work in 2022, contributing to SDGs 3 and 16 - Uruguay performed very well in remote education and will serve as an example to other countries, but it is necessary to increase efforts so that all children and adolescents can take advantage of this opportunity, contributing to SDGs 4 and 10 - UNICEF supported care systems for children in early childhood and their families, contributing to SDGs 1, 2, 3, 10 and 16 - UNICEF contributed to highlighting the link between gender-based violence and violence against children, with the aim of generating an integrated response from the State and contributing to SDGs 5 and 16/ - UNICEF’s first activities on the ground focused on improving the response to migrant families with children on the border with Brazil, contributing to SDGs 1, 2, 3 and 10 - Thanks to the generosity of donors in Uruguay and the innovativeness of the fundraising team, the number of donors continued to grow, contributing to SDG 17 |
| UNICEF Libya | Libya | National | Country Office Annual Report | 2 | **Capacity building initiatives** | - By 2021, Libya was struggling to meet many of its Sustainable Development Goals (SDGs). - Contributing to the realization of SDG 2 and 3, in partnership with the Ministry of Health, UNICEF focused on strengthening primary health care through a system-building approach to reduce neonatal, child, and maternal mortality and morbidity. - Contributing to the realization of SDG 6, UNICEF worked with national stakeholders to prevent and mitigate the impact of disease outbreak (including COVID-19 infection) by ensuring access to safe water, sanitation and hygiene, with a focus on the most vulnerable - Contributing to the realization of SDG 4, UNICEF works with national partners to improve access and quality of education. - Contributing to SDG 16, during the first part of 2021 and in the context of the newly established GNU, UNICEF focused on strengthening its partnership with government counterparts relevant to the development of a national child protection system - Contributing to SDG 1, in partnership with the World Bank, UNHCR, UNDP and WFP, UNICEF supported and contributed to critical dialogue on development of a national social protection policy. |
| UNICEF Tajikistan | Tajikistan | National | Country Office Annual Report | Child-related SDGs | **Health equity programmes** | - *Mid-term Development Programme (MTDP)* 2021-2025 was adopted and implemented by the government to outline commitment towards achieving the SDGs and key child-related priorities - Despite falling child mortality rates and significant improvement in the overall nutritional status of children in Tajikistan, undernutrition remains a critical public health concern – particularly in remote regions and rural areas. |
| UNICEF 2021 | Jordan, Myanmar, Ethiopia, Sudan, Tanzania | National | Report | 2, 3, 4, 5 | **Cash transfer programmes** | - *Ujana Salama*, a pilot project in Tanzania was one of the first to provide evidence of the effectiveness of a cash-plus intervention implemented within an existing government run social protection programme. |
| UNFPA, UNICEF 2020 | Bangladesh, Burkina Faso, Ethiopia, Ghana, India, Mozambique, Nepal, Niger, Sierra Leone, Uganda, Yemen, Zambia | National | Report | 5 | **Sexual and reproductive health programmes** - **Child Marriage** | - The rate of progress must be at least 8 times faster than the rate observed over the past decade in Bangladesh to meet the national target, or 17 times faster to meet the SDG target to end child marriage by 2030 - In Burkina Faso, 32,956 adolescent girls were supported by the programme to enroll and remain in primary and secondary school despite the disruptions in schooling due to the COVID-19 pandemic. - In Ethiopia, over 152,000 adolescent girls were reached through life-skills training, including comprehensive sexuality education, with information and skills on Sexual and reproductive health programmes, and legal and psychosocial services. - In Niger, more than 740 cases of child marriage have been postponed or cancelled, and 27,321 children have received child protection care services |
| UNICEF Bolivia 2019 | Bolivia | National | Country Office Annual Report | 1, 2, 3, 4, 5, 6, 10, 16, 17 | **Emergency resilience building** | - UNICEF Bolivia demonstrated its ability to plan and implement required rapid and appropriate responses considering the fluctuating national contexts, including landslides in La Paz, wildfires in Chiquitanía and the post-electoral social conflict. |
| UNICEF Malawi 2021 | Malawi | National | Country Office Annual Report | 6 | **Capacity building initiatives** | - UNICEF Malawi has tested, scaled and adopted several high-impact innovations in 2021 in close collaboration with young people. - Some of the key achievements on the innovation front included the *Intelligent Community Health Information System, and the work through the African Drone and Data Academy* which has equipped 440 young people from 25 African countries with 21st century skills. |
| UNICEF Namibia 2019 | Namibia | National | Country Office Annual Report | 2, 3, 4, 6 | **Capacity building initiatives** | - Against the backdrop of UNICEF custodianship of ten global SDG indicators, Namibia Country Office (NCO) supported advancement of the Sustainable Development Agenda for children using a lifecycle approach. - In the first decade integrated programming facilitates progress towards achievement of SDGs 2, 3, 4, and 6. - Interventions in the second decade considered the interdependence and indivisibility of human rights in a bid to progress towards Sustainable Development Goals 3, 4, 5 and 16. |
| UNICEF 2019 | Philippines | National | Report | 3, 4 | **Nutrition and food security** | - The Philippines has seen significant progress on reducing the prevalence of child wasting – from 8.1% in 2013 to 5.6% in 2018 – and is on track to achieve the global nutrition target for wasting. - The Department of Health generally attributes the decrease in wasting prevalence to (1) the use of locally generated evidence on implementation, (2) supportive leadership and (3) consensus around the importance of integrating services to treat children with SAM into the health system. |
| UNICEF 2019 | Ethiopia, Cambodia, Lebanon, Ghana, Liberia, Tajikistan, Sierra Leone, Nepal, Madagascar, Uganda, Lao PDR, Tanzania, Cameroon, Mali, India, Kenya, Siberia | National | Report | 3 (3.1, 3.2, 3.8), 6 | **WASH initiatives** | - Improved WASH services for 3.6 million care seekers. As of 2018, the program has reached 590 health care facilities in rural areas, comprising 20% of the total rural primary health care facilities in Madagascar |
| UNICEF Sierra Leone 2019 | Sierra Leone | National | Country Office Annual Report | 3.2 | **ECD** | - The health, nutrition and HIV results contributed to achieving national priorities under Clusters 1 and 2 of the MediumTerm National Development Plan (MTNDP); SDGs 2 and 3; UNICEF Western and Central Africa Regions Key Results for Children (KRC) 1 and 2; UNDAF Pillars 1 and 3; and Annual Management Plan (AMP) Priority 1 (integrated with Early Childhood Development (ECD)). |
| UNICEF Oman 2019 | Oman | National | Country Office Annual Report | 1, 2, 3 (3.2), 4, 5, 10, 16 (16.2) | **ECD** | - Setting up and piloting the cross-sectoral case management system as well as development of the IECD model. - Work on these models has proven to be an effective strategy in a high-income developing country, where the Government has resources to take them to scale. |
| UNICEF Jamaica 2019 | Jamaica | National | Country Office Annual Report | 1, 2, 3, 4, 5, 16 (16.2) | **Capacity building initiatives** | - In 2019, UNICEF Jamaica took a deliberate approach to combine communication, advocacy and programming efforts for a concentrated and sustained focus on violence against children over several months, |
| UNICEF China 2019 | China | National | Country Office Annual Report | 1, 2, 3 (3.1, 3.2), 4 (4.2), 16 | **Capacity building initiatives** | - UNICEF through its assistance to the Government of China continues to work towards realizing children’s rights in China while strengthening their engagement with GoC to advance the SDGs for children in other developing countries. |
| UNICEF | Ethiopia | Sub-national | Report | 6 | **WASH initiatives** | - The ONEWASH Plus programme is a catalyst for wider improvements in service delivery, knowledge management, and policy at national level - Programme financial management was based on zero cash advances to implementing partners, which has avoided related issues such as outstanding advances and programme implementation delays. It was possible because the main NGO partner (World Vision) was in a position to carry the initial costs themselves. |
| UNICEF 2020 | Kazakhstan | National | Document | 3, 4, 16, 17 | **Capacity building initiatives** | - Kazakhstan’s *Adolescent Mental Health and Suicide Prevention programme* has positively impacted adolescents nationwide as, for example, suicide rates for adolescents age 15-19 participating in the programme continues falling. - The AMHSP programme shows that an intersectoral approach to creating a data-driven mental health response, awareness-raising, capacity- building, evaluation and advocacy are all necessary to change stigma and systems so that adolescents can claim their rights to a healthy life free from harm. |
| UNICEF 2019 | Bosnia and Herzegovina. | National | Country Office Annual Report | NS | **ECD** | - The country is now better able to use Developmental Behavioural Scales to detect developmental delays in children and provide basic early childhood interventions (ECI). - This contributed to the provision of quality ECD services to over 2,000 children and their families. In addition, over 110 parents gained enhanced skills on ECD/ECI through parenting education provided in two cities. |
| UNICEF 2020 | Cambodia, DRC, Ghana, Lao, Liberia. Papua New Guinea, Siberia, Timor Leste | National | Mixed methods – Global report | 3, 6 (6.1, 6.2) | **WASH initiatives** | - Climate-smart WASH solutions delivered to health care facilities and local communities in tandem save - Climate change provides a catalyst for taking action and making WASH improvements that save costs and contribute to wider resilience and national carbon emission reduction goals. |
| UNICEF 2019 | Turkmenistan | National | Report | 3, 4 (4.2) | **ECD** | - The capacity development programme, run in cooperation with the Ankara University of Turkey, continued, resulting in additional PHC facilities being able to provide early identification and intervention services. 7,000 children were assessed using the new tool in selected areas in 2019; 8 per cent needed early intervention services. |
| UNICEF 2019 | India | National | Country Office Annual Report | 3 (3.8) | **Health equity programmes** | - Routine data and unpublished reports show improvement in all indicators along the continuum of care for *RMNCH+A.* |
| UNICEF 2019 | Nepal | National | Country Office Annual Report | 6 | **Capacity building initiatives – Maternal and child health** | - At local level, UNICEF with national NGOs supported 24 municipalities to prepare three-year costed plans for maternal and child health |
| UNICEF 2019 | Pakistan | National | Country Office Annual Report | 6 (6.1, 6.2) | **Capacity building initiatives – Maternal and newborn health** | - UNICEF supported the development of a strategic accountability framework for maternal and newborn healthcare, contributing to 40,000 sick newborns that received medical care and 1,150 preterm babies managed in 17 kangaroo-mother care centres. - The scale-up of home-based newborn care, kangaroo care and possible serious bacterial infection treatment in seven districts enhanced quality and improved survival for 399,299 newborns. |
| UNFPA-UNICEF | Bangladesh, Burkina Faso, Ethiopia, Ghana, India, Mozambique, Nepal, Niger, Sierra Leone, Uganda, Yemen, Zambia | National | Mixed methods – survey and interviews | 5.3 | **Health equity programmes** | - Through the Global Programme support, more than 3,000 established adolescent clubs provided life-skills-based education to 70 per cent of the adolescent girls in targeted programme areas. - Empowering Girls through education support: 500 of the most marginalized out-of-school adolescent girls received on-the-job informal apprenticeship training with theoretical and life-skills training and finally linked with job opportunities. Over 80,000 girls in programme areas have been supported to enroll and remain in school through support leveraged from other programmes. |
| UNICEF Tunisia 2020 | Tunisia | National | Country Office Annual Report | 1, 2, 3, 4, 6, 16 | **Emergency resilience building** | - Clearly identifying and communicating gaps in the realization of child rights in line with the SDGs and its indicators framework offered an important entry point, easy to communicate to all stakeholders and aligned to the national SDG policy agenda. - A light review of the policy dialogue methodology to integrate the risks of the COVID-19 pandemic across the different areas ensured the continued relevance of the document following the crisis. |
| UNICEF Bangladesh 2019 | Bangladesh | National | Country Office Annual Report | NS | **Sexual and reproductive health programmes** - | - Over 69,000 sick newborns made a full recovery in 32 UNICEF-supported special care newborn units, while 1,295 low birthweight babies received KMC in 36 other UNICEF-supported facilities – twice as many as received KMC in 2018. - Prevention of mother-to-child transmission (PMTCT) of HIV interventions were extended to 12 hospitals, with 62,000 women receiving HIV counselling/testing during antenatal care. |
| UNICEF South Africa 2020 | South Africa | National | Country Office Annual Report | 3.2 | **Capacity building initiatives – health systems** | - Strengthening of national and sub-national health systems capacities for evidence-based planning and monitoring of a comprehensive package of EMTCT, maternal, newborn and child health and nutrition services with focus on identifying and covering the unreached; (2) Capacity strengthening of national and sub-national education systems for the delivery of quality and inclusive education; - All these were adopted to improve South Africa's ability to meet SDG 20230 deadlines |
| UNICEF Niger 2019 | Niger | National | Country Office Annual Report | 2 (2.2), 3 (3.2), 4 (4.2, 4.5), 5 (5.3), 6, 16 (16.9) | **Capacity building initiatives – social sector** | - There was significant progress at policies and systems level in key social sectors with potential impact towards achieving the SDGs as well as key results for children - Niger continued to make progress with structural economic reforms, which underpin Niger’s continued economic growth estimated at 6.3% in 2019 compared to 6.5% in 2018. |
| UNICEF Rwanda 2019 | Rwanda | National | Country Office Annual Report | NS | **Capacity building initiatives – multisectoral programs** | - The presence of UNICEF in Rwanda has led to the implementation and integration of several policies and programs that aim at meeting SDG target goals, especially in relation to education, health service delivery, WASH, and social protection for children and youth in the country. - Funding from external partners like organizations (World Vision) and other governments (Japan) have supported the implementation and integration of key programs related to HHSDGs like access to nutrition. |
| UNICEF Tajikistan 2020 | Tajikistan | National | Country Office Annual Report | NS | **Capacity building initiatives** | - To support the government in achieving its national SDG targets, the joint programme "Financing SDGs in Tajikistan" was initiated in partnership with UN Women and UNDP. - The programme supports realization of national priorities, leveraging additional resources through an SDG financing roadmap and more efficient budget utilization |
| UNICEF Syria 2019 | Syria | National and Subnational | Country Office Annual Report | 2 (2.9), 4, 5 (5.3), 16 (16.3) | **Emergency resilience building** | - After years of stagnation, slow progress was recorded towards SDG 2.9. Progress was recorded towards SDG4, as enrollment in schools increased. - Efforts were made concerning SDGs 16.3 and 5.3 to reduce crimes of violence against children, criminalizing the recruitment of children and adopting national programs for the rehabilitation and integration of children. |
| UNICEF Nepal 2020 | Nepal | National and Subnational | Country Office Annual Report | NS | **Emergency resilience building** | - COVID-19 pandemic negatively impacted progress towards H&HrSDGs in Nepal. - Adaptation was required and demonstrated by external organizations like UNICEF to meet outcomes like reduction of child stunting, reducing violence against children, social protection etc. |
| UNICEF South Sudan 2019 | South Sudan | National and Subnational | Country Office Annual Report | 2 (2.2), 3, 4 (4.1, 4.2), 6, 16.2 | **Nutrition and food security** | - Reduced prevalence of global acute malnutrition among children in 2019 (23 percent in 2010, 13 per cent in 2018) and of child stunting (31 per cent in 2010, 17 per cent in 2018). |
| UNICEF Dominican Republic 2019 | Dominican Republic | National | Country Office Annual Report | 2 (2.2), 3 (3.1, 3.2), 5, 16.9, | **Capacity building initiatives** | - Several programs implemented by UNICEF with multi-sectoral collaboration to make progress towards H&HrSDGs. - Lessons learned from implementation highlight the important role of people’s behavior |
| UNICEF India 2020 | India | National | Country Office Annual Report | NS | **Emergency resilience building** | - COVID-19 pandemic curtailed progress made towards achieving H&HrSDGs, and impacted some regions more than others |
| UNICEF Algeria 2019 | Algeria | National | Country Office Annual Report | 1, 3, 5, 8 | **Capacity building initiatives** | - Despite the prevailing political context in 2019, capacity building efforts helped to revitalize partnerships with line ministries such as MoE and MoNS. - In the context of the SDGs, strengthening national systems through the application of RBM for inclusive social development was relevant. |
| UNICEF 2019 | Haiti, South Sudan, Ethiopia, Lebanon, Israel and Palestine, Somalia, Jordan | National | Report | 6 (6.1-6.2) | **WASH initiatives** | - The multiple actions carried out so far across the WASH and health sectors align with the Government of Haiti’s National Plan for the Elimination of Cholera in Haiti 2013–2022 - The implementation of the Global Compact on Refugees is helping the Ethiopian government to resolve long-term humanitarian crises, support peaceful coexistence between refugees and host communities, and realize its obligations to meet the SDGs for all of its people. |
| UNICEF 2021 | Ghana, Indonesia, Jordan, Malawi | National | Report | 4, 16 (16.2) | **Sexual and reproductive health programmes – adolescent pregnancy, child protection** | - Interventions allowed to create a national, regional and district cadre of 120 trainers on Safe Schools and to identify and mobilize school-level champions for safe schools. - A total of 1,200 administrators learnt about the provisions of the education sector response to adolescent pregnancy. The GES has banned the use of corporal punishment in school. - The percentage of children who experience verbal or physical violence according to the survey has shown a steady and considerable decrease from 44.8 per cent (verbal) in 2009 to 15 per cent in the academic year 2018-2019 and from 40.3 per cent (physical) to 8 per cent for the same period. |
| World Bank 2019 | Vietnam | National | Qualitative Report | 2, 6, 10, 14, 15, 16, 17 | **Infectious disease management** | - Significant gaps still remain with AMR implementation among countries. - This report finds that countries can make AMR-related investments more efficient and cost-effective by improving technical capacity and data quality, enhancing inter- agency coordination and building public awareness of the AMR challenge. - The report also urges the development community to go beyond technical solutions that focus exclusively on the misuse of antimicrobials. There needs to be more focus on interventions that cut across multiple sectors like improving public health systems, increasing access to clean water and sanitation, building resilient agriculture and food systems, educating younger generations on AMR, or designing urbanization and infrastructure to stop contamination |
| IDS - Gu et al, 2020 | Kenya, SriLanka | National | Qualitative Report | 17 | **Capacity building initiatives** | - The trilateral partnership between the countries (UK, China) is key to achieving SDG 17 for developing measurements of progress on sustainable development especially in developing countries |
| UNDP | Indonesia | National | Qualitative Report | 3, 17 | **Infectious disease management** | - From January to September 2022, there were 8,042 patients with confirmed cases of drug-resistant tuberculosis (DR-TB), and approximately only 54% of them received treatment, likely because of the barriers posed by the range of economic and social challenges associated with DR-TB. - Technological advances in health have also been adapted to support COVID-19 vaccine delivery in Indonesia, facilitating the administration of 429 million vaccine doses and the full vaccination of 171 million people (as of July 2022) |
| UNDP | Vanuatu | National | Qualitative Report | 5, 16 | **Sexual and reproductive health programmes –GBV** | - Vanuatu women’s movements have demonstrated the integral role women-led organizations play in mobilizing men and women in GBV prevention and   response, given that they are closer to the grass roots and are well grounded in advocacy.   - They have led advocacy work on GBV prevention and response with the communities and government, leading to a policy framework on GBV. |
| UNDP | Fiji, Iceland, Uzbekistan, Togo, South Africa, Cabo Verde, Canada, Egypt, Finalnd | National | Qualitative Report | 5, 8, 10 | **Emergency resilience building** | - *Novissi* in Togo has been heralded as a unique example of extending protection to uncovered populations while simultaneously building social protection system capacity. If leveraged properly, the newly created Novissi database can become a critical tool to respond to future shocks. - Free COVID-19 testing, a comprehensive tracking system and, later, widespread vaccination allowed Iceland to maintain low infection rates and avoid lockdowns, thus minimizing the situational risk of increased violence within the home. |
| UNDP | Bosnia and Herzegovina, Cambodia, El Salvador, Jordan, Kyrgyzstan, Lao PDR and Cambodia, Madagascar, Niger, Paraguay, Philippines, Tajikistan, Togo | National and Subnational | Qualitative Report | 6, 16 | **WASH initiatives** | - The activities carried out will contribute to the achievement of the national development programme, which is aligned with the SDGs and in keeping with the logic of ensuring universal and equitable access to affordable water, equitable access for all to adequate sanitation and hygiene services, and an end to open defecation, with special attention paid to the needs of women and children. |
| UNDP | Pakistan | National | Report | NS | **Cash transfer programmes** | - The Emergency cash scheme helped raise social protection coverage from 18.1% in 2017 to 54.7% in 2020. - More than 4.6 million women received cash payments in the category reserved for women. In the remaining categories, there were 8 million female and 6.8 million male beneficiaries. 26 beneficiaries were transgender. - Despite these achievements, the programme met only a fraction of beneficiaries’ needs and left out millions of people. |
| UNDP | Papua New Guinea, Turkmenistan, Cameroon | National | Qualitative Report | 1, 17 | **Emergency resilience building** | - In the midst of supply chain shortages, UNDP’s ability to quickly, efficiently, and transparently procure essential emergency medical supplies for frontline healthcare workers was critical to saving lives during the global COVID-19 pandemic. |
| UNDP | Albania, Armenia, Bangladesh, Colombia, Jordan, Mongolia, Mozambique, Tanzania | National | Qualitative Report | 5, 16 | **Sexual and reproductive health programmes - GBV** | - The UPR has had a significant impact on gender equality and women’s rights and gave an opportunity for better alignment, coordination and implementation of recommendations from Treaty Bodies and Special Procedures. |
| UNDP | Indonesia, Philippines, Thailand, Vietnam | National | Assessment Report | 3 | **Sexual and reproductive health programmes – HIV, contraceptives** | - The CSE program which was implemented had successes in creating awareness for antiretroviral therapy (ART) for HIV and around condom use in schools. - However, challenges arose in the quality of the rollout, its evaluation, and the lack of funding allocations by other local governments to support the programme. |
| UNDP | India | National | Report Brief | 3 | **Capacity building initiatives** | - *Electronic Vaccine Intelligene Network* has ‘saved’ 90 million vaccine doses, reduced stock-outs by 80 percent and produced an estimated future return on investment of nearly 300% |
| UNFPA 2019 | South Africa | National | Qualitative | 3, 5 | **Health insurance schemes** | - The adoption of a primary health-care package has helped to ensure the inclusion of many essential SRHR interventions, although services delivered at both secondary or tertiary level and outside the health sector have been omitted and in total, 281 conditions relating to SRHR are captured in the *South Africa Health Benefits.* |
| UNFPA | Benin, Sudan, Uganda, Zambia, Bangladesh, Togo | National | Qualitative | 3 (3.1) | **Capacity building initiatives** | - Capacity building efforts for *EmONC, midwifery and fistula* were credited with improving access to skilled birth attendance. - The tuition payments to sponsor midwives were not sustainable as they have not been mainstreamed into government budgets. |
| UNFPA 2020 | Bangladesh | National | Qualitative | 5,3 | **Sexual and reproductive health programmes** | - Several challenges were identified with the implementation of *Girl Shine* and the other interventions implemented at the Rohingya Camps in Bangladesh |
| Rahman 2021 | Bangladesh | National | Qualitative | All | **Capacity building initiatives** | - Out of the 17 goals, Bangladesh has achieved 8 goals (goal-1, 2, 4, 5, 7, 8, 9, 17). Bangladesh lags in achieving SDG 3, 6, 8, 10, 11, 12, 13, 14, 15, and 16 - Public-Private Partnerships and external funding are needed to close gaps |
| Feng et al, 2022 | China | National | Impact Analysis | 4, 5, 10, 16 | **Emergency resilience building** | - A 1% increase in the SDG4 score is associated with a 0.115% reduction in daily new confirmed COVID-19 cases on average. - A 1% increase in the SDG5 score is associated with a 0.0411% reduction in daily new confirmed COVID-19 infections on average |
| **Updated Search (March 2024) = 28 studies** | | | | | | |
| Agyemang et al, 2023 | Ghana | Facility level | Descriptive, cross-sectional | 3, 9 | **Digital health initiatives** | - 10% of healthcare facilities have adopted the use of *Lightwave e-health information management system* for health records. - LHIMS deployment has enhanced service efficiency. - Work experience and computer literacy significantly influenced efficiency. |
| Sejie et al, 2023 | Botswana | Community-level | Multiple case | 3 | **Community-based health programmes** | - *National Tuberculosis and Leprosy Program* is facilitated by the presence of national and district policies and SOPs for community TB care. - The operational effectiveness of the CTBC approach is compromised due to various barriers, including inadequate funding, lack of training, and insufficient supervision. |
| Buckley et al, 2023 | Ireland | Facility-level | Stakeholder analysis | 3 | **ECD** | - Public Health Nurses, were identified as fundamental partners in achieving SDGs in a disadvantaged Irish community. - The multidisciplinary *Kidscope* model, a novel interagency pediatric clinic offering free developmental assessment and onward referral for children aged 0 to 6 years, improves childhood developmental delay and addresses healthcare exclusion. |
| Duvendack et al, 2023 | India | National | Case study | 1, 5, 8 | **Health equity programmes** | - *Linking the banking system with women’s self-help groups (SHG)* through Bank Sakshi’s have the potential to change attitudes towards digital financial inclusion. - Research suggests that financial inclusion is an enabler for 7 of the 17 SDGs (World Bank 2014). - The program requires better support and infrastructure to enhance its impact on women’s financial inclusion. |
| Prieto-Egido et al, 2023 | Peru | National | Case study | 1, 2, 3, 4, 5 (5.b), 7 (7.2), 8, 9, 10 (10.2), 11 (11.a) and 17 | **Digital health initiatives** | - *Mayu’s activities in telemedicine, eHealth tools and digitization of public services in rural areas* are found to affect only a few SDGs (SDGs 3, 9, and 17). However, connectivity opportunities could be extended to other areas such as education (SDG 4), agriculture (SDG 2), or governance (SDG 16). - Recommendations include flexible regulatory frameworks for RMIOs, collaboration with community networks, increased public support in developing civil infrastructure (e.g. towers), and fostering multi-stakeholder partnerships to enhance service delivery in rural areas |
| Anju et al, 2023 | India | Subnational | Qualitative | 3 | **Health equity programmes** | - The 25-year history of *decentralized healthcare administration* in Kerala indicates both successes and failures. - Recommendations include providing central support without disempowering local governments, enhancing local decision-making spaces, improving technical collaboration, and maintaining the balance between local autonomy and state/national priorities. |
| Handebo et al, 2023 | Ethiopia | National | Cross-sectional | 3 | **Health insurance schemes** | - Nearly four out of 10 (43.2%) reproductive-age women in Ethiopia enrolled in *community-based health insurance scheme* - Reproductive-age women aged 20 and 34 years, rural residents, and women living in the Somali, Benshangule Gumuz, SNNP, and Gambella regions, women affiliated with Protestant religion, women with large family size, female-headed households, and those with more than five living children were associated with low CBHI enrolment. |
| Khalid et al, 2023 | Pakistan | National and sub-national | Innovation and Practice Report | 3 | **Health equity programmes** | - By seizing the opportunity presented by the SDG3 *GAP platform*, development partners have engaged with the Ministry of NHSR&C on UHC reforms in a more coordinated manner, and the Ministry has played a critical role in steering technical analyses and strategic dialogues and engaging partners as per their comparative advantages. - Achieved development of a national health financing framework, the provincial adaptation of EPHS and launching the National Health Support Program for implementing EPHS at the sub-national level. |
| Aunger et al, 2023 | Tanzania | National | Innovation and Practice Report | 6 | **WASH initiatives** | - *The Nyumba ni Choo programme,* a *Community Led Total Sanitation approach to stop Open Defecation* (OD) found significant increases in improved toilet coverage from 43% in 2016 to 72% in 2020 |
| Penggalih et al, 2023 | Indonesia | National and sub-national | Qualitative | 2, 8 | **Cash transfer programmes** | - Provision of *village funds (Dana Desa*) significantly improved infrastructure and economic activities in rural areas by allocating 60-100% of funds. - Disproportionate emphasis on infrastructure over human capital investments. |
| Charway et al, 2023 | Ghana | National, regional, and district levels | Case study | 3 (3.3) | **Community-based health programmes** | - Increased participation in *sports and health programs* with impacts on community health were found. - Policy goals are well-defined, but implementation is hindered by resource distribution and non-involvement of local actors. |
| Lehman et al, 2024 | (Iran) EMRO comprises 21 MemberStates and the occupied Palestinian territory. | National and regional | Situational analysis | 3 | **Community-based health programmes** | - *National community health worker (CHW) programs* are essential for achieving UHC and SDGs, but face challenges like governance issues and funding sustainability. - CHW programmes play an increasingly important role in primary health care in the Eastern Mediterranean Region, providing promotive, preventive, and emergency services. - Integration into health systems is crucial for success |
| Narayan et al, 2023 | India | National | Cross-sectional | 1 (1.1, 1.2, 1.3), 2.2, 3, 5 | **Cash transfer programmes** | - Simultaneous participation in *various social protection programs* lowers women’s morbidity by at least 25%, but women’s BMI increases only in states implementing those programs well. For children, there is no robust evidence of impacts. |
| Pradhan et al, 2023 | India | Sub-national (three states: Bihar, Chhattisgarh, Odisha) | Qualitative | 1, 2, 3, 5 and 10 | **Cash transfer programmes** | - Women perceive themselves as more empowered through involvement in the *SWABHIMAAN intervention program*, which also improved the community's and their households' nutritional status |
| Veda et al, 2024 | India | Sub-national (state of Manipur) | Commentary | 3 (3.8), 10 | **Cash transfer programmes** | - A health assurance scheme, the *Chief Minister-gi Hakshelgi Tengbang (CMHT)* enrolled 6,81,648 beneficiaries of about 30 lakh population in the state and treated 132,733 beneficiaries. - Service utilization indicators of some key national programs, namely, tuberculosis (TB), HIV/AIDS, and childhood diseases, show a mixed performance. - Data from NSS and NFHS show progress in health service utilization and maternal and child health indicators. |
| Gupta et al, 2023 | India | Sub-national (state of Manipur) | Spatial analysis | 1, 2, 3, 4, 5, 6, 8, 9, 10, 13, 14, 15, 16 | **Cash transfer programmes** | - *Mahatma Gandhi National Rural Employment Guarantee Act (MGNREGA),* a public welfare scheme contributes to multiple SDGs beyond SDG 1, including SDGs 2, 3, 4, 6, 9, 13, and 15, with significant spatial variation in asset distribution across districts |
| Aguila et al, 2023 | Philippines | Sub-national (municipalities of Bay and Calauan, Laguna) | Quasi-experimental | 17 | **Nutrition and food security** | - *Pinggang Pinoy Kid’s Plate program* emphasizes government-industry-academia alliance to achieve SDG 17 - The triad partnership between government, industry, and academia improved the nutritional status and knowledge, attitude, and behavior of school children and their mothers. - The number of underweight children shifted to normal nutritional status by 25.3% after 120 feeding days. |
| José Grin et al, 2023 | Brazil | Subnational (City of Jundiai) | Case study | 11 | **Urban Planning** | - Integration of SDGs into urban policies has led to improved urban planning and sustainability in *Jundiaí.* |
| Askelrod et al, 2024 | United Kingdom, Nigeria, Iran | National | Case study | 3 (3.4) | **Product regulations – salt and fat content** | - The UK achieved significant progress in reducing population salt intake (a 7% reduction in salt intake from the period 2005–2019 from 8.1 to 7.5g/day). - In Iran, the maximum permitted levels of saturated and trans fatty acids in food were modified in the national regulations. |
| Venter et al, 2023 | South Africa | Sub-national (Province: Limpopo) | Case study | 3.6 | **Road safety measures** | - *The Limpopo programme* is rooted in successful public and private partnerships and the coordination of targeted interventions and actions aimed at reducing mortality on Limpopo roads. |
| Venancio et al, 2023 | Brazil | National | Case study | 3.2 | **Nutrition and food security** | - The integration of SDGs into national nutrition policies has led to improvements, but challenges remain in terms of funding and implementation |
| Tesema et al, 2023 | Ethiopia | National | Mixed methods | 3 (3.4, 3.8) | **NCD promotions** | - The research demonstrates a lack of progress in providing integrated NCD services due to gaps in the core strategic and operational levers of PHC |
| Curry et al, 2024 | India | National | Book | 2.2 | **Nutrition and food security** | - Recommendations include prioritizing actions to promote complementary feeding with a focus on gender equity. It was stressed that for promoting appropriate complementary feeding, there was a need to focus on behavior change interventions and appropriate use of food supplements. |
| Stern et al, 2022 | Iraq and Lebanon | National | Case study & Program Evaluation | 5 (5.2), 8, 16 | **Sexual and reproductive health programmes - GBV** | - Adapting the prevention program to country-context through participatory action - The pilots in Iraq and Lebanon suggest that UNDP’s *“Indashyikirwa” project* offers promise for adaptation as well as integration into livelihoods programming, including in crisis settings. |
| Curry et al, 2024 | India | National and sub-national | Case study & Policy Brief | 16 | **Capacity building initiatives** | - In the last year, UNDP India helped initiate three new *SDG coordination centers* in partnership with state governments, indicating the continued relevance of the framework. - There is substantial and growing knowledge on SDG localization in India. The Indian experience is also noteworthy as it attempts to bridge the gap between SDG visioning and the reality of on-the ground implementation |
| RTI International, 2019 | Cabo Verde | National | Case study & Investment Case Analysis | 1, 3 (3.4), 8, 10 16 | **Product regulations - tobacco** | - The investment case shows that there is an opportunity to reduce the social and economic burden of tobacco in Cabo Verde. - Current tobacco tax is 11.2% of the retail price - Recommendations include adopting comprehensive tobacco control legislation, raising tobacco taxes and engaging the tourism sector as a leader in tobacco control |
| Ministry of Public Health, Madagascar, 2019 | Madagascar | National | Case study & Investment Case Analysis | 3, 16 | **Product regulations - tobacco** | - Current tobacco tax is 80.4% of the retail price - Enacting the four FCTC tobacco-control measures would avert significant economic losses and save lives. |
| UNDP and UNU-IIGH, 2020 | Indonesia, Peru, Republic of Moldova | National and sub-national | Case study & Program Evaluation | 1, 3, 4, 5 (5.2), 8, 11, 16 | **Sexual and reproductive health programmes - GBV** | - Local action plans were developed, and village think tanks for community action - Midline study undertaken in Peru in May 2021 reported progress in raising community awareness of GBV as a problem and of service referral pathways. |
| **Updated search (January 2025) = 24 studies** | | | | | | |
| Ferronato et al, 2024 | Kenya, Malaysia | National | Review | 6, 11 (11.6), 12 (12.3, 12.4, 12.5), 14 (14.1) | **WASH initiatives – Waste Management** | - In Kenya, the selective collection of plastic waste brings economic resources, as well as access to basic services, involving the informal sector and providing appropriate infrastructures in remote areas (SDG 1.4) and increases the amounts of MSW collected and managed in controlled facilities (SDG 11.6). - With respect to plastic waste minimization and recycling, circular actions effectively addressed SDG 12.4, 12.5, together with SDG 11.6. Furthermore, the adopted methods to minimize plastic waste and recycling lead to sustainable management and efficient use of natural resources (SDG 12.2). |
| Sornpaisarn et al, 2024 | Thailand | National | Narrative review & stakeholder consultations | 3, 17 | **NCD promotions** | - Evidence showed that the Thai government fully adopted the multi-sectoral collaboration approach; however, a major complex obstacle – implementation failure – has hindered the Thai government’s attempt to execute multi-sectoral action plans to combat NCDs as recommended by the WHO |
| Chen et al, 2024 | China | National | Modelling study | 1, 2,3, 6, 7, 9, 11, 12, 13, 14, 15.17 | **Emergency resilience building** | - By 2060, compared with the Reference pathway, the average scores for five environmental SDGs including clean water and sanitation (SDG6), affordable and clean energy (SDG7), responsible consumption and production (SDG12), climate action (SDG13), and life on land (SDG15) were improved by 5.43, 15.69, 32.22, 51.13, and 8.60 points on average. - In addition, mitigation policies improved the performance against four socioeconomic SDGs including no poverty (SDG1), zero hunger (SDG2), industry, innovation and infrastructure (SDG9), and sustainable cities and communities (SDG11), by 10.47, 8.70, 7.63, and 20.02 points on average by 2060. |
| Mamta et al, 2024 | India | National | Quasi-experimental (pre-post interventional) study | 3 (3.1) | **Sexual and reproductive health programmes – Birth Preparedness and Complication Readiness** | - Further studies should explore the awareness of BPCR at the individual and community levels, as their involvement is elemental to achieving SDG 3, target 1 of providing safe motherhood and reducing maternal mortality. |
| Forbat et al, 2024 | Switzerland | National and sub-national | Programme assessment | 3,5,7,8,17 | **Community-based health programmes - Health Promotion** | - From an empirical perspective, results obtained by Lausanne, and based on the analytical grid comprising 6 thematic issues and 56 categories explicitly comparable to the 169 SDG targets, tend to emphasize the global achievement of policies relevant both in terms of health promotion and sustainable development. |
| Tolla et al, 2024 | South Africa | National | Qualitative study | 3 (3.7),5, 10 | **Sexual and reproductive health programmes – contraceptive services** | - Adolescent girl and young women’s use of contraception services is dependent on the behavior of health care providers, which is dependent on attitudes and skills of health care providers, as well as improvements in their working conditions. Focusing interventions on these factors may help to bring South Africa closer to achieving the SDGs. |
| Moughalian et al, 2024 | India | National | Cross-sectional study - secondary data | 3,5 | **Sexual and reproductive health programmes – contraceptive services** | - Increasing access to modern contraception is a core component of universal health coverage and is essential in meeting Sustainable Development Goals by 2030. Results show an encouraging picture of the ASHA programme in increasing the uptake of modern contraceptive methods in India. |
| Yang et al, 2024 | Pakistan | National | Retrospective | 3 (3.8.1, 3.8.2) | **Health insurance schemes - UHC** | - Despite the mounting challenges of economic underdevelopment and endemic disease burdens, Pakistan’s policy initiatives in primary care provision, along with the provincial governments’ efforts in piloting and expanding UHC programs, have yielded a consistent increase in the UHC index. |
| Verma et al, 2024 | India | National | Retrospective | 16 | **Health equity programmes** | - The positive correlation between institutional deliveries and birth registration presents a clear pathway towards achieving universal birth registration and targeted health & social welfare policies in India. |
| Akther et al, 2024 | India | National and sub-national | Survey-based analysis | 3, 6,11 | **WASH initiatives – urban waste management** | - Availability of optimum waste management, sanitation and public health services, forms core of an urban development plan, and lies at heart of the UN SDG framework. |
| Ottie-Boakye et al, 2024 | Ghana | National | Cross-sectional mixed methods study | 3 | **Health insurance schemes** | - Despite the progress made by Ghana in policy formulation on achieving universal health coverage, especially, for older persons, there remains a coverage gap with the implementation of these policies - NHIS enrollment among older persons is 60%, with gender, occupation, self-rated health, and healthcare utilization being key determinants. Perceived barriers include cost, technological challenges, and inadequate service quality. |
| UNCESCO and UNICEF, 2024 | Ghana | Global –National case studies | Annual Global Results Report | 4 (4.2) | **ECD** | - Scale up of play-based kindergarten with training of teachers is expected to transform learning outcomes for 1.2 million kindergarten children across the country, supporting Ghana to accelerate achievement of SDG Target 4.2. |
| UNFPA and UNICEF, 2024 | Burkina Faso | Global –National case studies | Annual Global Results Report | 5 (5.3) | **Sexual and reproductive health programmes –Ending FGM** |  |
| UNICEF, 2024 | Bolivia | Global –National case studies |  | 2, 3 | **Health equity programmes** | - UNICEF is supporting member states to localize the SDGs by setting ambitious national targets for children, establishing systems to track progress in reducing inequality, and ensuring that ‘no child is left behind’. Bolivia depends on $218,268 of UNICEF Thematic Funds and should explore increasing financial commitments to SDGs |
| UNICEF, 2024 | Nigeria | Global –National case studies | Annual Global Results Report | 4 (4.1, 4.4, 4.6), 5, 8 | **ECD** | - To tackle the learning crisis, UNICEF’s educational strategy will continue to support children and adolescents through their three pivotal learning transitions: early childhood education to prepare them for formal schooling; fundamental learning to ensure proficiency in literacy, numeracy, and other basics; and skills development to equip every young individual with a comprehensive set of transferable, digital, occupation specific, and entrepreneurial skills, enabling them to thrive in both life and work |
| UNICEF, 2024 | Global | Global –National case studies | Annual Global Results Report | 5 (5.2, 5.3), 16 (16.2, 16.3, 16.9) | **Health equity programmes** | - The mid-term review of implementation of UNICEF’s Strategic Plan has identified key areas and priorities to accelerate progress in Goal Area 3 for the remaining Strategic Plan cycle, and towards the SDGs |
| UNICEF, 2024 | Ghana, Nigeria | Global –National case studies | Annual Global Results Report | 6, 13 | **Emergency resilience building** | - There's a notable emphasis on integrating climate resilience into WASH initiatives, with significant progress made in developing climate rationales across multiple countries - Prioritizing joint sector reviews to diagnose sustainability issues affecting WASH services, coupled with strategic capacity-building programs for local governments are recommended in Nigeria |
| UNICEF, 2023 | Brazil, Nigeria | Global –National case studies | Annual Global Results Report | 1, 10 | **Health equity programmes** | - Increased investment by EUR 99 million for child protection and equity initiative and intersectoral approaches are needed to address child poverty and food security challenges in Brazil. - Nigeria increased investments in social services to address child poverty. |
| UNICEF, 2023 | Kenya, Bangladesh | Global –National case studies | Annual Global Results Report | 2, 3 | **Community-based health programmes** | - Primary health care system strengthening including training programs for community health workers across 47 counties is expected to reduce maternal and child mortality in Kenya. - Primary health care and immunization efforts with expanded Digital health initiatives platforms for monitoring of doses have led to improved child health outcomes. |
| UNICEF, 2024 | India, Rwanda | Global –National case studies | Global report | 4,5, 10 | **ECD** | - Early Childhood Care and Education access remains unequal, with significant rural-urban disparities in India. Increase investment in workforce development and expand outreach programs are recommended - Improved enrolment but persistent gaps in Early Childhood Care and Education service quality. Enhanced investment in infrastructure and workforce development are recommended in Rwanda |
| UNICEF, 2023 | Ethiopia, Pakistan | Global –National case studies | Global results report | 3, 5 | **Health equity programmes** | - Gender disparities in education and maternal health in Ethiopia are improving through policy reforms and targeted programs with increased public sector funding for gender-responsive programming with additional support from development partners. - Expanding the Lady Health Workers Programme in Pakistan via increased budget allocation has significantly improved women's healthcare access especially in rural areas. |
| UNDP, 2022 | Mongolia, Nigeria | Global –National case studies | Global results report | 8, 17 | **Capacity building initiatives** | - Public-private partnerships are crucial for sustainable financing reforms in Mongolia due to limited domestic financial resources and dependency on international funding. - Nigeria's Integrated National Financing Framework provides a structured approach to mobilizing SDG-aligned investments. |
| UNDP, 2023 | Moldova | National | Pilot intervention study | 5, 16 | **Sexual and reproductive health programmes –GBV services** | - Despite government allocation of 36 million lei in 2014 for GB response, GBV services remain underfunded; community-based interventions show promise in addressing gaps including increasing shelter availability and strengthened intersectoral collaboration |
| UNFPA, 2024 | Rwanda, Bangladesh | National | Annual global health report | 3, 5 | **Capacity building initiatives** | - Midwifery education expansion via a mobile mentorship initiative has improved maternal and newborn health outcomes in Rwanda. The program will be further upgraded by integration with a mobile application to facilitate simulated practice - Bangladesh must enhance data reporting and response mechanisms for maternal mortality. |
| Legend:- GBV: gender-based violence, HrSDGs: Health related Sustainable Development Goals, NCD: Non-communicable diseases, NS: Not specified, SDG: Sustainable Development Goals, UNDP: United Nations Development Programme, UNFPA: United Nations Population Fund UNICEF: United Nations International Children’s Emergency Fund, RTI: | | | | | | |

## Supplemental Table 5: Challenges reported by included studies to implementing the SDGs pre-COVID, during COVID and post-COVID

| Domains | Pre-COVID Challenges (July 2019 – Feb 2020) | COVID Challenges (March 2020 – May 2023) | Post-COVID and Ongoing Challenges from updated review (June 2023 – onward) |
| --- | --- | --- | --- |
| Political and Enhanced Financial Commitment | | | |
| Political Commitment | - Lack of clear, aligned and updated national policies.^55 166^ - Delays in policy and legislative reform are prevalent, due to political sensitivity or weak coordination mechanisms.^55 166^ - Weak inter-sectoral and multi-level government coordination significantly hinders effective implementation of child and adolescent rights policies^77^ - Underinvestment and lack of domestic financial commitment to HHSDGs despite economic growth.^88^ | - Political and policy focus shifted country priorities to COVID-19 response and management, disrupting progress in HHSDG-related reforms, including sexual and reproductive health rights, child marriage laws and youth and gender initiatives. ^149 167 168^ | - In Kyrgyzstan, the health sector integrates SDGs and indicators into Health 2030 and follows an intersectoral, whole-of-government approach to policy development and implementation. However, COVID-19 revealed weaknesses in the primary healthcare system due to low levels of political and financial investment.^31^. - COVID-19 placed significant strain on the public health system, and has undone years of progress made by national governments, which was compounded by polycrises including polio outbreaks, floods, landslides, earthquakes, border conflicts and other natural disasters.^75 169^ - The COVID-19 crisis emphasized that short-term responses are ineffective, especially in the contexts with insufficiently developed social services systems.^169^ - National policies do not conceptualize and articulate the integration of CHW programmes into health systems as part of comprehensive primary health care ^170^ |
| Financial Commitment | - Core public services such as WASH, ECD and waste management were underfunded.^69 70 162^ - Some countries faced high out-of-pocket health spending with minimal public investment (e.g., Bangladesh spent only 0.76% of GDP on health while households took on 72% of costs.^144^ - Political shifts disrupted funding commitments, with a notable emphasis on donor dependency and fragile public financing structures.^77 168^ - Insufficient domestic resource allocation and financial planning was a barrier to humanitarian response.^69 162^ | - Emergency pandemic spending diverted resources away from HHSDG priorities, exposing weak fiscal resilience in many countries.^171^ ^172 173^ - Heavy reliance on donor funding.^171^ - Service delivery was disrupted due to funding gaps and workforce reallocations in healthcare delivery, education and social protection sectors.^17 79 174^ - Vulnerable populations were unable to access financial assistance available during the pandemic.^175^ | - Political unrest led to the bank accounts of government partners being frozen resulting in difficulties with disbursing necessary funds.^17^ - Budgets and timelines for projects had to be extended till 2025/26 due to the delays and inability to complete project activities as a result of the COVID-19 pandemic.^176^ - Financial procurement from external organizations like UNICEF prove to be essential for countries to meet HHSDG targets.^169^ |
| Institutional set-up and capacity building | | | |
| Institutional set-up | - Absence of centralized systems and departmental coordination mechanisms were a barrier to systematic planning and oversight.^166^ - Weak child protection and cross-sectoral collaboration limited a coordinated national response to support families and children.^56 115^ | - Pandemic responses disrupted service delivery and strained institutional structures, particularly in maternal, neonatal health and child protection sectors.^79 174^ - Fragile education systems led to long-term closures of schools. ^79 139^ - Institutional capacity to address violence against women and girls during the pandemic were weakened.^171^ | - Despite the existence of national strategic plans to combat NCDs, a lack of follow-through and implementation was observed despite the engagement of non-health stakeholders.^177^ |
| Capacity Building | - Health worker capacity limited due to low knowledge levels, legal barriers for refugees and inconsistent training standards.^69 81 117^ - Fragmented education across health training institutions.^69^ - Outdated infrastructures in facilities.^69^ | - Healthcare workers were overburdened by increasing demand for COVID-related services, leading to an increase in infections, mental health issues (i.e. anxiety, depression, sleep problems and distress). This led to reduced availability and motivation of skilled staff. ^30 31 173 174^ - Pandemic control measures such as travel restrictions, physical distancing, restrictions on group gathering, mask mandates and handwashing disrupted program implementation^30 72 73 139 178^ | - Additional training needed for CHWs to increase program effectiveness.^179^ |
| Monitoring and Evaluation | | | |
| Monitoring & Evaluation | - National monitoring and evaluation systems were outdated and not aligned with SDGs, making the tracking of targets difficult. ^21 69 83^ - Limited availability and access to high-quality and timely data.^80 83 142 150^ - Weak national statistical systems and a lack of standardized data collection procedures reduced the reliability of data.^69 134 180^ | - The pandemic disrupted in-field data collection due to reprioritization of government M&E capacity.^139^ - Chronic disease monitoring was limited by health system strains and PPE shortages, revealing gaps in preparedness^31^ - M&E efforts were significantly impacted by the pandemic due to factors such as PPE shortages, health system strain, missing infectious disease indicators, and movement restrictions.^31 139 149^ | - The COVID-19 pandemic revealed that the Healthy Cities Initiative in China did not include many indicators for infectious diseases, exposing gaps of the initiative.^149^ - Due to redirected priorities as a result of the pandemic, not much energy was reserved for upper-level government to focus attention on other aspects of the Healthy Cities Initiative.^149^ |
| Collaboration and Partnerships | | | |
| Stakeholder engagement | - Hierarchical systems limited open dialogue and collaboration between stakeholders.^69^ - Private sector engagement required careful attention and negotiation.^70^ | - COVID-19 control measures restricted international partner coordination, delaying education and community engagement activities.^139 167^ - School closures and the ban on public gatherings put a pause on in-person stakeholder activities (e.g. community dialogues).^139^ | - Roles of various stakeholders were not clear at first compelling the State government to issue orders to make role clarifications.^181^ - Decentralizing administrative control of health institutions was met with resistance from doctors and professional medical associations.^181^ |
| Multi-sectoral coordination | - Weak coordination across ministries, partners and institutions lead to duplication of efforts or a delay in adopting unified approaches ^69 77^ - Implementation of humanitarian aid models by NGOs created tensions due to refugee populations receiving better infrastructure than host communities supported by governments.^70^ | - Pandemic pressures weakened inter-ministerial coordination, contributing to fragmented and delayed service delivery. ^92^ | - COVID-19 has increased the need to strengthen sectoral and multisectoral coordination, including among international actors, made it more vital to prioritize the most vulnerable, and to push for greater ownership by the government on developmental achievements.^75^ |
| Health Equity | | | |
| Improving health equity | - Marginalized populations (e.g. children with disabilities, poor and displaced communities, adolescents), faced a great risk of exploitation, violence, health inequities due to systematic barriers and environmental crises (e.g. droughts).^68 77 113 143^ - Issues of GBV and structural discrimination (e.g. underrepresentation of women and criminalization of drug use) reduced access to care and led to poorer health outcomes.^77 143 182^ | - The pandemic exacerbated existing health inequalities with internally displaced individuals and women and children, facing increased risks of FGM and reduced access to essential services.^31 139^ - The closure of schools and termination of community programs due to COVID-19 limited adolescent access to health, protection, and mental health services. ^139 171 183^ - Domestic physical and psychological violence surged during pandemic lockdowns.^139 171 183^ | - Mass displacement due to conflict and recurrent droughts and other compounding factors have exacerbated risks of female genital mutilation (FGM) especially in areas where FGM is a precursor to marriage, which is seen to provide economic security. - Disruption of social protection for children including – schools, peer network and community support systems. - Interrupted education due to school closures with ongoing effects even after the peak of COVID-19. |
| Adapted Interventions and Information Exchange | | | |
| Adapted Interventions and Information Exchange | - No evidence of challenges pre-COVID reported in included studies. | - International consortiums faced challenges made worse by the pandemic with regards to establishing relationships, communication and coordination with development partners.^167^ | - Information available to MoH and shared with other countries in the region about their community health worker (CHW) programmes is uneven and limited due to lack of integration into health systems.^170^ |
| Acronyms: Health and Health-related Sustainable Development Goals (HHSDGs), Sustainable Development Goals (SDGs), Community Health Worker (CHW), Early Childhood Development (ECD), Gross Domestic Product (GDP), United Nations Children's Fund (UNICEF), Universal Health Coverage (UHC), Accredited Social Health Activists (ASHAs), Monitoring and Evaluation (M&E), Personal Protective Equipment (PPE), Gender-Based Violence (GBV), Female Genital Mutilation (FGM), Noncommunicable Diseases (NCDs), Organizations of Persons with Disabilities (OPDs), Non-Food Items (NFIs), Voluntary National Reviews (VNRs), World Health Organization (WHO), United States Agency for International Development (USAID), European Union (EU), Primary Health Care (PHC), Mobile Health (MHealth), Non-Governmental Organization (NGO), Civil Society Organization (CSO), Ministry of Health (MoH), United Nations Educational, Scientific and Cultural Organization (UNESCO), Good Governance for Medicines (GGM), Electronic Vaccine Intelligence Network (eVIN), Sistem Monitoring Imunisasi Logistik secara Elektronik (SMILE), Low- and Middle-Income Countries (LMICs), Access and Delivery Partnership (ADP). | | | |

## Supplemental Table 6: Strategies reported by included studies for implementing the SDGs pre-COVID, during COVID and post-COVID

| **Domains** | **Pre-COVID Strategies (July 2019 – Feb 2020)** | **COVID Strategies (March 2020 – May 2023)** | **Post-COVID and Ongoing Strategies from updated review (June 2023 – onward)** |
| --- | --- | --- | --- |
| **Political and Enhanced Financial Commitment** | | | |
| Political commitment | - Governments integrated the SDGs into national development plans, including national frameworks, strategies and reports.^55 68 82 113 137 143 154 182 184^ - National reporting processes like Voluntary National Reviews (VNRs) were undertaken by governments to share the country’s experiences, challenges and lessons learned on the implementation of the 2030 Agenda. ^55 115 154^ - National strategies placed special emphasis on vulnerable groups (e.g. children and refugees), with a focus on equity.^69 77 82 117 137 154 161 166^ | - COVID-19 resources were used as a strategic opportunity to leverage investment in strengthening routine systems and programmes including immunization, oxygen supply, newborn care and infection, prevention and control services in health facilities.^185^ - The establishment of inter-ministerial committees to reduce the impact of COVID-19 on informal workers, which included a workers’ emergency benefits fund. Committees would develop policies, collect data and coordinate initiatives to assist informal workers whose livelihoods had been impacted by COVID-19.^171^ - Countries have shown a commitment to achieving the SDGs by establishing and implementing national policies, strategies, and plans that align with and integrate the SDGs.^173^ - Legislative measures provide a legal framework for policy implementation and enforcement on specific SDG issues (e.g. FG< access to health services).^72^ | - Ownership of sanitation programs were assigned to the highest levels of governments to instill a sense of accountability and commitment among national and local leaders, government officials, development partners and private sector partners personally, to ensure they were aligned with the core mission of the programme.^167^ |
| Financial commitment | - SDG-related initiatives were supported by external donors such as UNICEF, WHO, USAID and the EU through direct funding or advocacy efforts. ^55 68 77 80 82 88 115 134 142-144 154 162 186^ - Establishment of investment cases for the achievement of UHC (SDG 3.8)^143^ - Internal resources were allocated by countries through national budgets to co-finance or independently fund SDG-aligned initiatives.^115 134 142-144 150 166^ ^115 134 142-144 150 166^ - The development of national guidelines with multi-billion dollar budgets to improve access to water and sanitation.^80^ | - Emergency or pandemic-related budget reallocations by national governments to alleviate burden on vulnerable populations. ^72 135^ - UNICEF evidence generation and advocacy in response to COVID-19 contributed to increased budget allocations to social sectors.^138^ - Under national COVID-19 response plans, ministries of health succeeded in mobilizing over millions of dollars in funding from national governments and donor sources to improve WASH services. ^132^ - UN entities leveraged SDG acceleration funds. ^185^ | - Increased flexibility in funding and implementation to adapt quickly to emergency situations and deliver timely results.^139^ - Leveraging crisis-driven political will for long-term investments in vulnerable populations – for e.g., multi-year commitments to early childhood education in Rwanda and South Africa.^135^ - Create integrated, joint financing frameworks aligned with national SDG target to leverage additional resources through an SDG financing roadmap and more efficient budget utilization.^169^ - To tackle resource limitations, the Consolidated WASH Account pool fund has grown, with new donors contributing, and the duration of the Phase 2 pool fund has been extended until mid-2024.^187^ |
| **Institutional set-up and capacity building** | | | |
| Institutional set-up | - Institutional arrangements focused on expanding UHC coverage through integrating services in PHC.^80 161 188^ - Reforms and restructuring of institutional arrangements to better align with the SDGs. ^142 143 162^ | - COVID-19 efforts were integrated into existing SDG structures.^86 135 138^ ^86 135 138^ - New institutions or technical units were created for program implementation.^141^ ^87 88^ ^87 88 139^ - Ministries established intersectoral oversight teams to support and monitor project implementation.^15 72 74^ | - The government has institutionalized the SDGs within its policy frameworks, notably through the development of a budgeting and tracking system called "Hyperion" by the Ministry of Finance which ensures that budget allocations across ministries and local government bodies reflect SDG priorities.^189^ |
| Capacity Building | - Training programs were implemented to strengthen health workforce and institutional delivery systems.^44 55 69 77 137 144 154 161 162^ | - Remote learning and digital tools were leveraged to continue programmatic activities and disseminate COVID-19 information.^33 139 190^ - Training of front-line workers was pivotal in pandemic response measures.^17 139 185^ - Emphasis on strengthening existing health system infrastructures in COVID-19 response^74 75 183^ - UNICEF agencies played an important role in improving emergency response, building resilience and emergency surveillance, upgrading the skills of government and NGO staff who are essential in COVID-19 prevention. Civil protection committees were also established at community level to effectively use available predictive and early warning data.^185^ - Intergenerational dialogues, education and social mobilization sessions were leveraged to ensure sustainability of interventions against FGM. This included engaging with community members, such as religious and community leaders to challenge social norms related to FGM.^17^ | - Human resource capacity development by UNICEF country offices for coordination and management of emergencies - Build and utilize technology and MHealth for monitoring maternal and child health, especially in the absence of in person consultations - Strengthen healthcare systems to improve responsiveness and achieve UHC integrating NCD prevention and control services within the PHC system and localizing implementation of national health priorities in district health offices.^173^ - Strengthen local and national systems for youth participation by implementing specific policies, institutional mechanisms and building capacities of adult stakeholders to support young people’s participation in a meaningful and safe way.^191^ - Strengthen community engagement through trusted local leaders – engage traditional and religious leaders in advocacy on health and education issues (e.g., returning pregnant girls to school, promoting safe schools). - Use mass media to disseminate health and rights information – Leverage radio and television in multiple languages to expand outreach on health measures, child protection, and COVID-19 prevention. - Promote inclusive education and right-age enrolment – As a foundation for improving long-term health and wellbeing outcomes. - Leverage digital platforms for awareness and engagement – Use online campaigns to raise awareness on child health, safety, and protection issues. - Integrate child protection themes into public health messaging – Addressing child labor, trafficking, and marriage alongside disease prevention supports broader SDG 3 targets.^33^ |
| **Monitoring and Evaluation** | | | |
| Monitoring & Evaluation | - Countries developed, implemented or strengthened indicator frameworks and monitoring tools to track HHSDG progress.^68 80 115 137^ Countries leveraged national statistics bureaus to support national SDG M&E. ^56 80^ | - COVID-19 data was integrated into existing monitoring platforms. ^13 139^ - Studies were commissioned to assess and analyze COVID-19 impacts on GBV, due to the increased vulnerability of women and girls during the pandemic and the need to maintain and strengthen prevention and assistance services for survivors of GBV and FGM. - Remote monitoring of programmes and resource mobilization aimed at sustaining continuity of essential services were scaled up by UNICEF as part of their COVID-19 response.^185^ - Surveys and administrative data were used for tracking of HHSDG outcomes.^17 111 130^ | - A protection monitoring and incident reporting (PMIR) system was developed in Nepal post-COVID-19 with the aim of determining if and how the current COVID-19 situation has created new protection risks and/or intensified preexisting protection issues. The protection monitoring system has enabled protection actors to identify key protection issues and needs and to advocate with the broader humanitarian community and the authorities for the continuation of priority protection activities.^72^ - Organizations of Persons with Disabilities (OPDs) in Myanmar also led monitoring of the effectiveness of assistance by measuring awareness and implementation of COVID-19 preventive measures; post-distribution monitoring for non-food items (NFIs); and follow-up on rehabilitation sessions through phone surveys.^74^ |
| **Collaboration and Partnerships** | | | |
| Stakeholder engagement | - Engagement of NGOs and community groups was pivotal in SDG implementation planning, delivery and monitoring.^44 55 69 70 80 88 94 113 162 166 180 186 192^ - Stakeholder consultations were conducted to improve implementation plans and garner political commitment.^44 83 88^ - Special engagement with universities and academic institutions for policy and program design, implementation and evaluation. ^88 113 115^ | - CSOs and NGOs were important leaders in COVID-19 response measures.^36 139 193^ - Youth and women's groups contributed to local solutions (e.g., ^17 74 76^) - Private sector partnerships were established during the pandemic to facilitate the disbursement of financial and technical resources (e.g., ^183 194^) - Academic and research institutions supported with conducting national assessments.^88 168 195^ - The Parliamentary Research Department was supported to produce compelling evidence that is informing evidence-based advocacy, legislation, and budgeting in Uganda. The results of a study on teenage pregnancy conducted in 2020 led to a motion for a resolution of parliament urging government to develop and enforce policies and strategies to protect girls against escalating cases of teenage pregnancy and child marriage during and after the COVID-19 pandemic.^72^ - UNICEF in partnership with Canada, Global Partnership for Education and UNESCO continued to support the ministries of basic and secondary education to run schools safely despite the pandemic. The financial and technical support strengthened communication for COVID-19 prevention in schools and built capacity of administrators to manage cases of COVID-19 at school.^183^ | - After COVID-19-induced school closures, the inclusion of parent– teacher association members in the menstrual hygiene management training helped trigger menstrual hygiene management-related activities at the community level.^139^ - For WASH, new partnerships with the education sector helped promote and improve hygiene practices in the pandemic context and strengthened emergency contingency planning and development programming post-pandemic.^136^ - For a broader impact on subnational planning processes, UNICEF partnered with the Bolivian Federation of Municipal Associations to generate child-focused capacity-building processes for local planning in the country’s nine departments.^77^ |
| Multi-sectoral coordination | - Multi-sectoral coordination was leveraged for child protection services. This involved collaboration between government ministries, healthcare facilities, schools, hospitals child protection committees and community members.^56 137^ | - Health and education sectors aligned for coordination and continuity of service. ^73 74 139^ - Local governments, agencies and NGOs came together for program implementation.^181 196 197^ | - Nesting a multisectoral initiative under a primary government agency provided momentum for the initiative and created a culture of collaboration among involved stakeholders. This clear and centralized form of leadership facilitated the creation of professional networks across the different sectors.^198^ - A multi-sectorial approach involving various actors, including government ministries, National Sport Federations, and Sport for Development organizations, has been adopted to implement SDGs, particularly in areas like health, with a focus on using sport as a tool for community well-being and promoting healthy lifestyles.^189^ |
| **Health Equity** | | | |
| Improving health equity | - Policies and programs aimed to specifically reach vulnerable groups like women, children, migrants and refugees ^44 55 56 77 83 113 154^ | - High-risk groups were identified (e.g. people with disabilities) and placed on the priority list for COVID-19 vaccinations, resulting from an effective advocacy effort from the Disability Action Council of Cambodia, OPDs, NGOs and the United Nations.^74^ - UNICEF, working with government and NGO partners, implemented operational and strategic activities including expansion of the flagship social safety net programme adding a cash top-up of child grants to support the COVID-19 response.^183^ - During the pandemic, targeted messages were developed on topics such as child marriage, FGM and gender-based violence due to the increased risks for these practices during COVID-19. Information on where to seek support and services during lockdown were emphasized. ^72^ | - Promotion of digital financial inclusion for women to access banking services. A multi-sectoral approach involving central and state governments, public and private sector banks and NGOs was used to expand financial inclusion in rural areas.^199^ |
| **Adapted Interventions and Information Exchange** | | | |
| Country-Country exchange of resources and information | - A policy dialogue was facilitated by UNICEF at the Africa-China Poverty Reduction and Development Conference of the forum on China-Africa Cooperation.^56^ | - Virtual regional dialogues and peer exchanges were conducted during the pandemic period (e.g.,^17 35 200^) - An Adolescent Kit for Expression and Innovation was implemented across multiple countries and was found to be particularly important during the COVID-19 response as it allowed adolescents to continue learning and skill-building.^139^ - Successful implementation of programs such as conditional cash transfers, community schooling and GGM were adapted by other countries.^72 135 168^   - Good Governance for Medicines (GGM) Programme specifically sought to safeguard availability of medicines in participating countries by preventing corrupt practices at various levels of the medicines supply chain.^168^ - The experiences of Indonesia in repurposing eVIN and SMILE, respectively, for inventory management and delivery of COVID-19 vaccines offer valuable lessons for other countries. ADP has helped to facilitate the transfer of knowledge and technical know-how to other LMICs. The successful vaccination drives and high vaccination coverage, aided by the operational and systemic efficiencies provided by the digital systems, have greatly benefited Indonesia, and helped enable quick economic recovery.^95^ | - The UNICEF country office in Bolivia designed crisis helplines originally to address child protection issues during the pandemic and was scaled up to be a model of strong humanitarian-development nexus programming. Within the framework of regular and humanitarian programming, the Bolivian country office is participating in a joint effort with Paraguay and Argentina, monitoring UN agency responses.^136^ |
| Acronym list: Health and Health-related Sustainable Development Goals (HHSDGs), Sustainable Development Goals (SDGs), Community Health Worker (CHW), Early Childhood Development (ECD), Gross Domestic Product (GDP), United Nations Children's Fund (UNICEF), Universal Health Coverage (UHC), Accredited Social Health Activists (ASHAs), Monitoring and Evaluation (M&E), Personal Protective Equipment (PPE), Gender-Based Violence (GBV), Female Genital Mutilation (FGM), Noncommunicable Diseases (NCDs), Organizations of Persons with Disabilities (OPDs), Non-Food Items (NFIs), Voluntary National Reviews (VNRs), World Health Organization (WHO), United States Agency for International Development (USAID), European Union (EU), Primary Health Care (PHC), Mobile Health (MHealth), Non-Governmental Organization (NGO), Civil Society Organization (CSO), Ministry of Health (MoH), United Nations Educational, Scientific and Cultural Organization (UNESCO), Good Governance for Medicines (GGM), Electronic Vaccine Intelligence Network (eVIN), Sistem Monitoring Imunisasi Logistik secara Elektronik (SMILE), Low- and Middle-Income Countries (LMICs), Access and Delivery Partnership (ADP). | | | |

## Supplemental Figure 1: SDG publication trends from 2015-2024 using peer-reviewed data sources


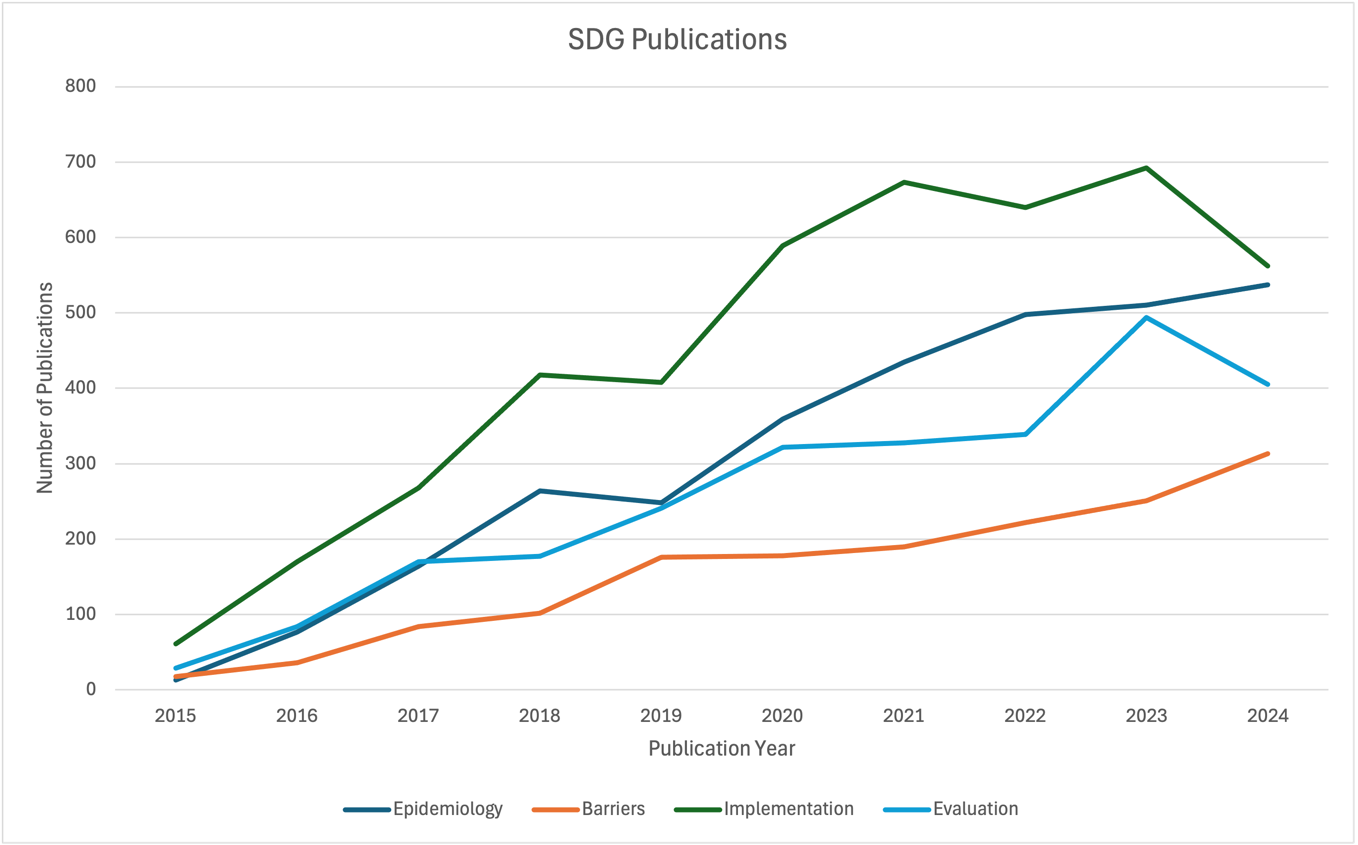


This figure illustrates the upward trend in SDG publications from 2015 to 2024. The keyword search terms used across databases are: "sustainable development goal" OR "sustainable development goals" OR SDG OR SDGs OR HHSDG OR "2030 agenda" OR "agenda 2030". These search terms were combined with each of the following terms separately to generate four trend lines: “epidemiology”, “barriers”, “implementation” and “evaluation”. This search was conducted on April 23rd, 2025. The list of databases searched to produce this trend data includes Embase Classic+Embase, CAB Abstracts, and Ovid MEDLINE(R) ALL.

## Supplemental Table 7: The 17 UN SDGs and their prevalence across included studies

| **SDG** | **Total Count** | **SDG** | **Total Count** | **SDG** | **Total Count** |
| --- | --- | --- | --- | --- | --- |
| SDG 1 | 25 | SDG 7 | 3 | SDG 13 | 4 |
| SDG 2 | 32 | SDG 8 | 16 | SDG 14 | 4 |
| SDG 3 | 109 | SDG 9 | 4 | SDG 15 | 3 |
| SDG 4 | 36 | SDG 10 | 16 | SDG 16 | 29 |
| SDG 5 | 43 | SDG 11 | 11 | SDG 17 | 15 |
| SDG 6 | 36 | SDG 12 | 4 |  | |

## Supplemental Figure 2: Number of studies grouped by intervention category


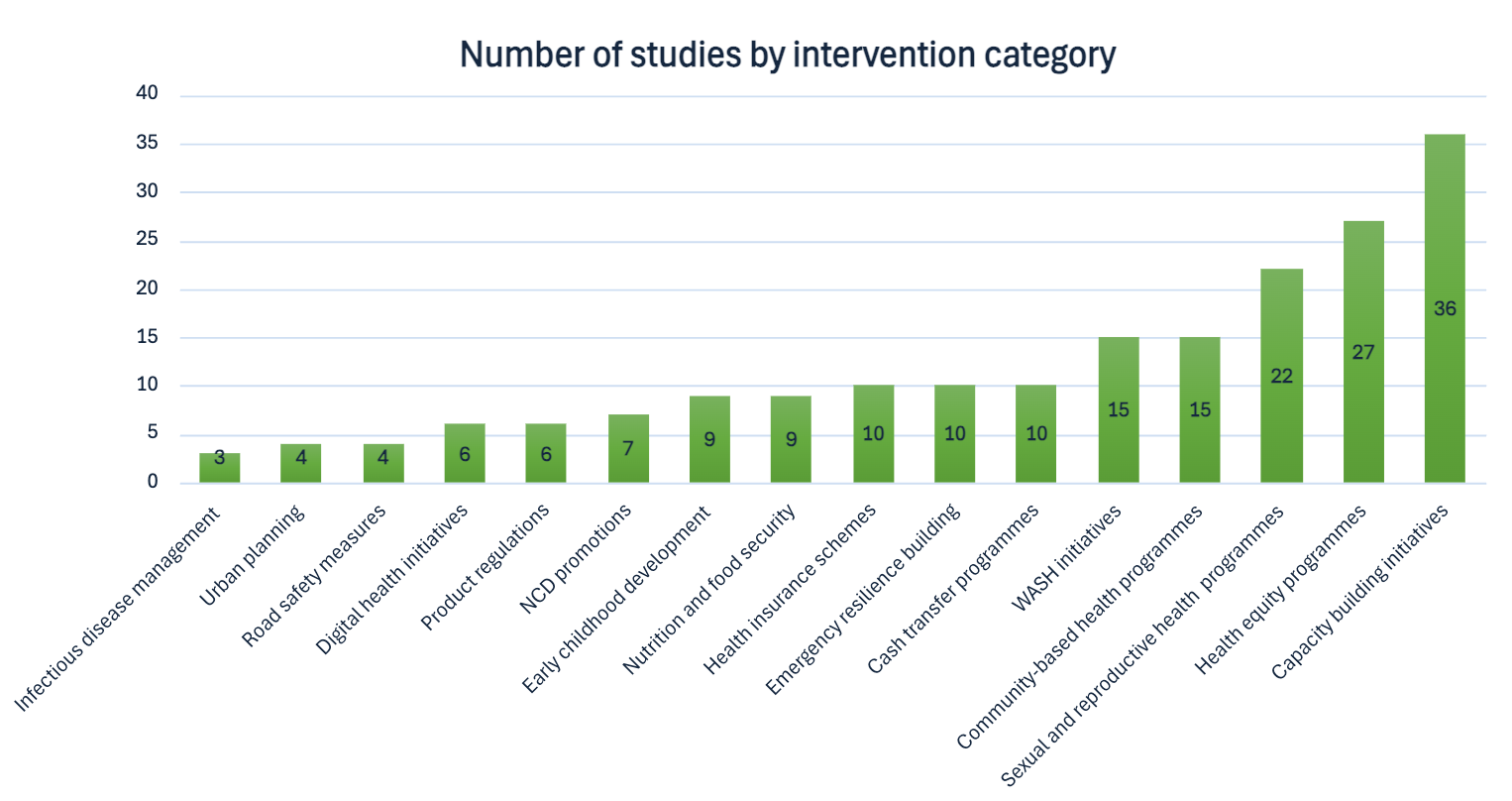


## Supplemental Table 8: Methodology for Figure 4: Availability of information about HHSDG implementation by sub-domain for all included peer-reviewed studies

| Figure 4 provides an overview of data availability across the six domains as reported in all included peer-reviewed studies. One reviewer went through the extraction sheet and coded availability of information as – 0=None (if study does not mention the domain), 1=Minimal (if study mentions the domain), 2=Some (if study reports on the domain with some level of information), and 3=Considerable (if study reports on the domain extensively). Information availability was assessed for each of the six domains for each of the 46 studies included. These scores were then totaled for each domain to calculate a percentage. Based on the percentages, the overall availability of information was again coded as minimal (</=30%), some (>30% and <50%) and considerable (>/=50%) and visualized in a radar plot whose outer bounds represent 100% (if all studies reported considerable information). |
| --- |
